# Insular routing to orbitofrontal cortex enables breathing awareness

**DOI:** 10.1126/sciadv.aeb3326

**Published:** 2026-07-29

**Authors:** Joshua Y. Assi, Stephan Bickel, Harly E. Greenberg, Ashesh D. Mehta, Thomas Similowski, José L. Herrero

**Affiliations:** ^1^ Department of Bioelectronic Medicine, Feinstein Institutes for Medical Research, Manhasset, NY 11030, USA.; ^2^ Departments of Neurology and Neurosurgery, Zucker School of Medicine at Hofstra Northwell, Hempstead, NY 11549, USA.; ^3^ Center for Biomedical Imaging and Neuromodulation, Nathan Kline Institute, Orangeburg, NY 10962, USA.; ^4^ Division of Pulmonary, Critical Care and Sleep Medicine, Department of Medicine, Zucker School of Medicine, Northwell Health, New Hyde Park, NY 11040, USA.; ^5^ INSERM, UMRS1158 Neurophysiologie Respiratoire Expérimentale et Clinique, Sorbonne Université, Paris F-75005, France.; ^6^ Groupe Hospitalier Universitaire APHP–Sorbonne Université, Hôpital Pitié–Salpêtrière, Département R3S, AP-HP, Paris F-75013, France.

## Abstract

How does the human brain detect and respond to disruptions in breathing? While animal studies have advanced our understanding of respiratory control, breathing distress in humans remains difficult to treat. It often arises not only from pulmonary lesions or brainstem dysfunction but also from how higher brain regions interpret breathing signals shaped by emotion and experience. We recorded intracranial cortical activity in neurosurgical patients during an interoceptive task involving transient breathing challenges. Conscious detection of these disruptions was predicted by early responses in the anterior insula, which routed signals to orbitofrontal and premotor cortices for appraisal and compensation. These cortical regions preferentially encoded inspiratory effort or airflow, revealing signal-specific processing that echoes functional segregation in brainstem centers. The present findings identify a dynamic insular-frontal circuit for sensing and adapting to respiratory challenges, offering insight into the neural basis of breathing awareness and its disruption in disease.

## Teaser

Cortical circuits track breathing effort and airflow to support awareness and adaptive responses to respiratory challenges.

## INTRODUCTION

Breathing is a basic life function, but its regulation depends on more than just brainstem circuits. In addition to rhythmic control by central pattern generators (*1*, *2*), higher forebrain regions actively monitor and modulate respiration (*3*–*5*). In rodents, cortical and subcortical projections to the pre-Bötzinger complex (preBötC) have been identified (*6*, *7*), and manipulating these pathways alters breathing patterns—causing hyper- or hypoventilation (*8*, *9*), sniffing (*10*), apnea (*8*, *11*, *12*), or cough (*13*). Whether these changes generate conscious breathing sensations or dyspnea remains unknown. In human research, individuals can report such sensations, but noninvasive methods (*14*–*17*) lack the precision to track cortical processing. As a result, how the forebrain interprets internal breathing signals remains unclear: Some patients fail to notice life-threatening respiratory compromise [e.g., asthmatics, opioid users, or acute COVID pneumonia (*18*–*22*)], while others report pathological breathlessness despite normal lung function (e.g., “dysfunctional breathing” associated with chronic anxiety, aging, or Long Covid) (*23*–*25*). These situations likely reflect disrupted cortical processing of ascending respiratory inputs [e.g., dyspnea (*26*)]. Understanding how the forebrain constructs conscious respiratory experience is essential to treating these disorders (*27*).

Leveraging rare human neurosurgical recordings and respiratory physiology, we tested the hypothesis that cortical processing of acute changes in respiratory mechanics is functionally segregated across regions. Specifically, we predicted that (i) the insular cortex would exhibit transient, stimulus-evoked activity, reflecting its role in detecting respiratory constraints; (ii) the orbitofrontal cortex (OFC) and fronto-motor cortex would show more sustained responses consistent with evaluative and compensatory motor regulation; (iii) these dynamics would unfold sequentially from insular detection to frontal modulation; and (iv) interindividual variability in detection performance would correlate with neural response strength in these regions.

To test these predictions, we directly probed respiratory perception using a novel inspiratory resistance detection task during intracranial electroencephalogram (iEEG) recordings in patients with epilepsy. This approach enabled millisecond-level tracking of respiratory signal flow across multiple cortical regions involved in interoception and breathing control.

## RESULTS

**Fig. 1.**
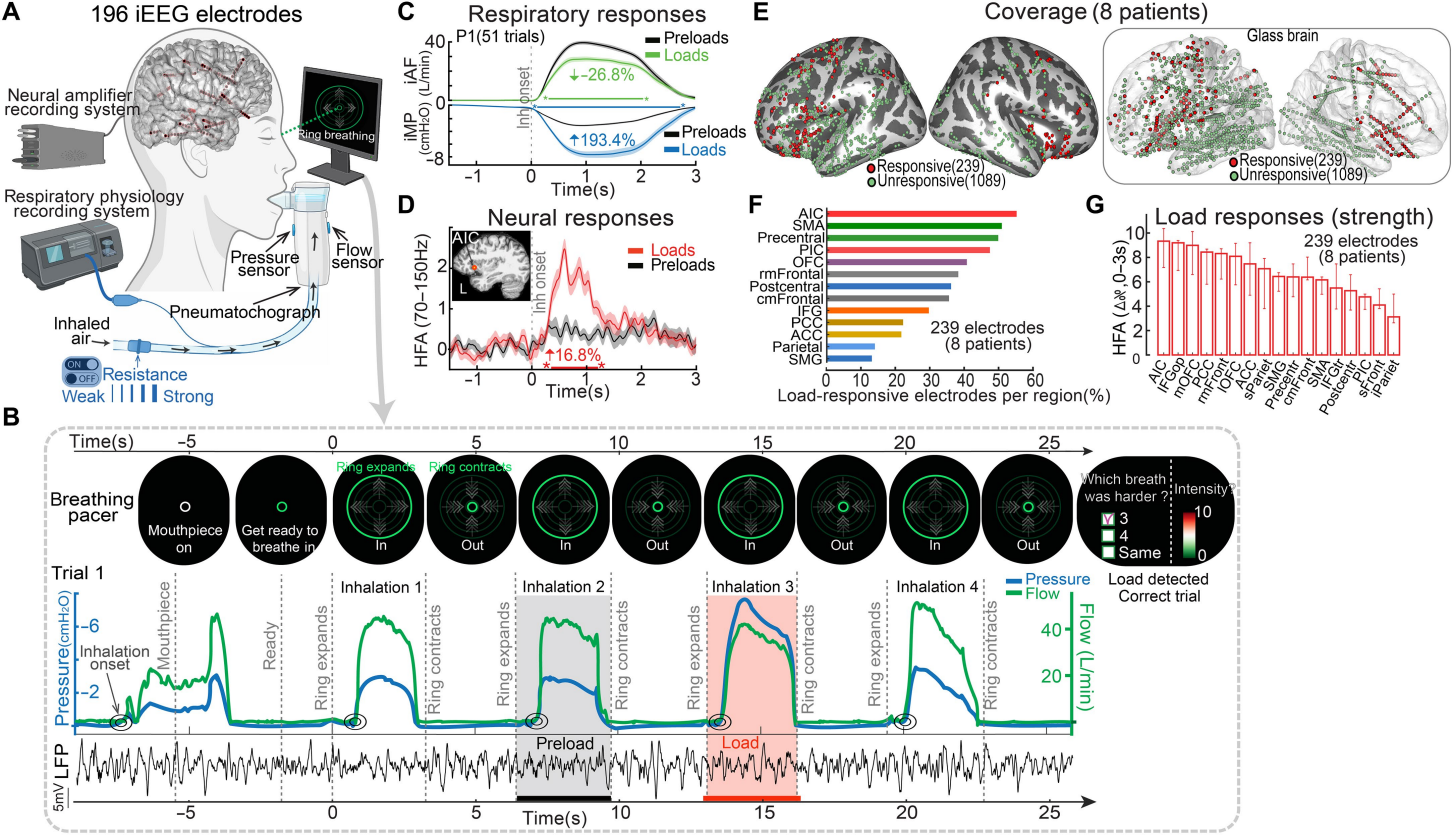
Intracranial recordings and respiratory responses during detection of inspiratory loads. (**A**) Simultaneous intracranial EEG and respiratory monitoring. Red dots show 193 stereo-electroencephalography (sEEG) electrodes implanted across cortical and subcortical regions in one patient. Participants breathed through a mouthpiece with nose clips while following a visual pacing cue (expanding/contracting ring). Inspiratory loads were applied using a two-way nonrebreathing valve. iMP and iAF were recorded with inline sensors. (**B**) IRDT. Participants took four paced breaths (3-s inhale and 3-s exhale) and detected a load applied randomly during one inhalation. An example trial shows effects of a 7.5 cmH_2_O liter^−1^ s^−1^ load on iAF, iMP, and AIC local field potentials (LFPs). L, liters. (**C**) Average respiratory traces from one subject across preloaded and loaded breaths (*n* = 51). Loads reduced airflow (iAF) and increased mouth pressure (iMP). (**D**) Example HFA (70 to 150 Hz) from a load-responsive AIC electrode. (**E**) Electrode coverage across all participants (*n* = 8) overlaid on the FreeSurfer average inflated cortical surface. Insets show corresponding glass-brain views (see fig. S1 for individual brains). Red dots indicate electrodes with significant load-evoked HFA increases (239 of 1328; 18%). (**F**) Percentage of load-responsive electrodes by brain region. (**G**) Neural gain across regions showing the magnitude of load-evoked HFA increases (median percent change ± interquartile range) during loaded versus preloaded inhalations (0 to 3 s postinhalation). mOFC, medial OFC; lOFC, lateral OFC; ACC, anterior cingulate cortex; PCC, posterior cingulate cortex; cmFrontal, caudomedial frontal; rmFrontal, rostromedial frontal; SMG, supramarginal gyrus; IFGop, inferior frontal gyrus pars opercularis; IFGtr, inferior frontal gyrus pars triangularis.

To examine how the cortex detects breathing constraints, we recorded iEEG from 11 patients with epilepsy implanted with electrodes across interoceptive brain regions during an inspiratory resistance detection task (IRDT; Fig. 1A). Three patients were excluded because of task noncompliance or excessive epileptiform activity (table S1), yielding 8 participants and 1328 electrodes for analysis (fig. S1). In each trial, participants breathed through a mouthpiece with a nose clip on while brief inspiratory resistances (loads) were unpredictably applied during specific inhalations (Fig. 1B). After each trial, they reported whether they detected a load, when it occurred, and how intense it felt. Load magnitudes spanned values above and below individual perceptual thresholds, while the respiratory rate and tidal volume were held constant across conditions (see fig. S2 and Supplementary Text for details).

### Respiratory loads alter breathing mechanics and are reliably detected

Inspiratory loads reduced airflow [inspiratory airflow (iAF)] and increased mouth pressure [inspiratory mouth pressure (iMP)] compared to preloaded (control) breaths (Fig. 1C). Across participants (*n* = 8), iAF decreased by 22.3% and iMP rose by 121.1% (*P* < 0.001; fig. S3, A and B), confirming effective load delivery and preserved respiratory responsiveness. Participants reliably detected suprathreshold loads, with detection declining near the perceptual threshold. Mean detection rates were 8.3, 32.2, 48.3, 75.9, 87.8, 91.1, and 100% (chance: 33%) for load magnitudes of 1, 2.5, 5, 7.5, 10, 12.5, and 15 cmH_2_O liter^−1^ s^−1^, respectively (fig. S3, C to E). False alarms were rare (1.4%). Individual detection thresholds, defined as the lowest magnitude detected on at least 50% of the trials, ranged from 2.4 to 9.6 cmH_2_O liter^−1^ s^−1^ (mean across subjects: 5.4). Lower thresholds were consistently associated with higher detection accuracy across individual participants (fig. S3, C to E). These behavioral metrics are aligned with prior normative data in healthy individuals (*28*, *29*) and with normal spirometry profiles (table S2) (*30*).

### Respiratory loads increase neural activity in interoceptive regions

Inspiratory loads reliably increased high-frequency activity (HFA; 70 to 150 Hz) relative to preloaded (control) inhalations in 18% of all electrodes (239 of 1328 across eight patients; Fig. 1, D to G, and fig. S1). Most load-responsive sites were in insular and frontal cortices. The example anterior insular cortex (AIC) electrode in Fig. 1D showed a 16.8% HFA increase (0 to 3 s postonset; *P* < 0.001), with effects beginning ∼285 ms after inhalation onset and lasting ∼1.5 s. Additional examples are shown in fig. S4. Across participants, the AIC had the highest proportion of responsive electrodes (53%; Fig. 1F) and the strongest HFA gains (median ΔHFA = 9.2%; *P* < 0.001; Fig. 1G).

### Conscious detection of respiratory loads amplifies neural responses in the anterior insula

**Fig. 2.**
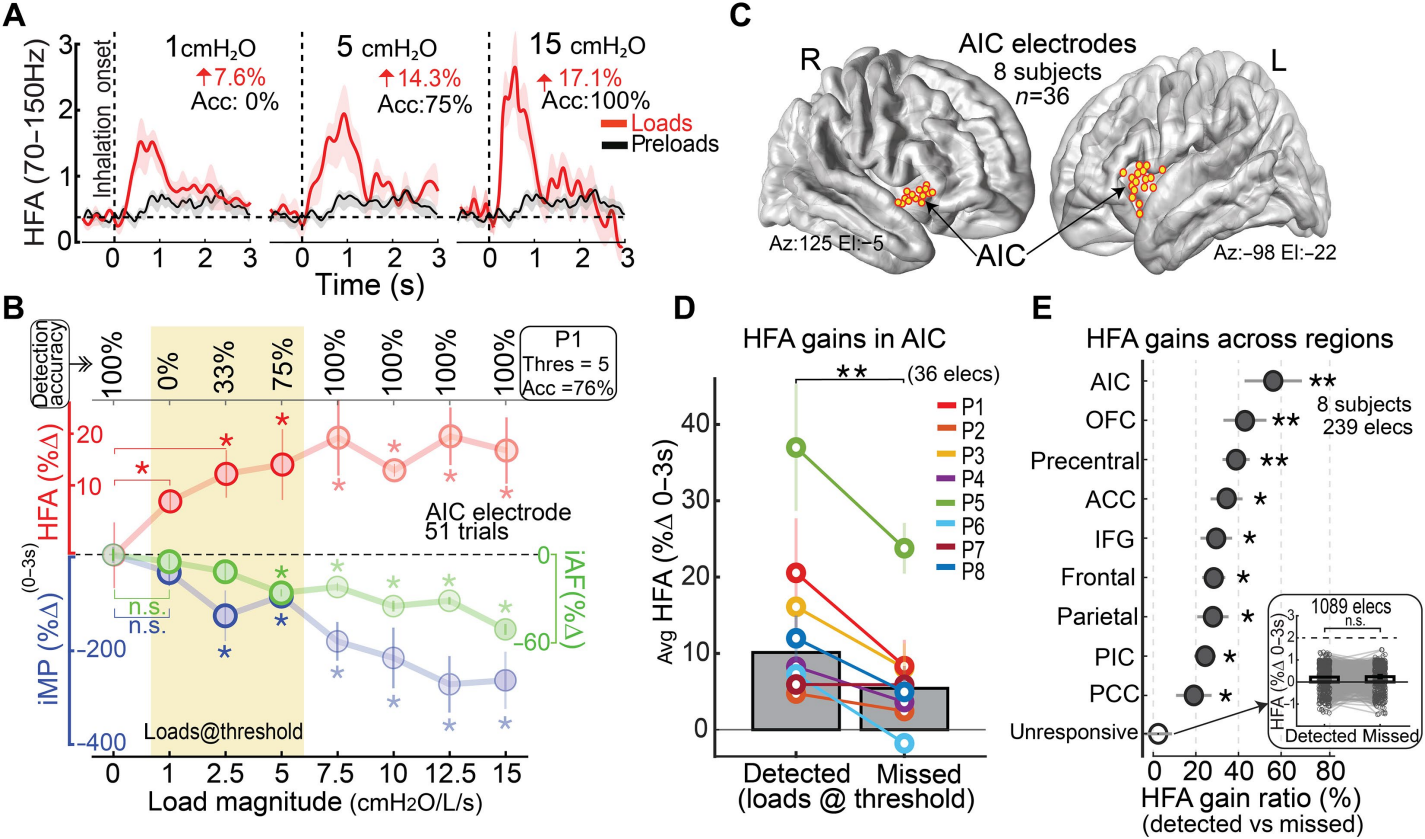
Anterior insular responses track conscious detection of inspiratory loads. (**A**) Magnitude-dependent responses at a single AIC site. Trial-averaged HFA responses from the same AIC electrode shown in Fig. 1D across three load magnitudes (eight trials each). HFA increased with load strength. (**B**) Physiology and detection behavior across load magnitudes. Load-related changes in airflow (iAF), pressure (iMP), HFA, and detection accuracy from the same participant, averaged over a 0- to 3-s window (means ± SE). Stronger loads increased HFA and detection rates while reducing iAF and increasing iMP. The yellow box highlights near-threshold magnitudes. Asterisks indicate significant differences from preload (magnitude: 0). n.s., not significant. (**C**) Electrode localization: AIC electrodes showing load-evoked HFA increases (*n* = 36, eight patients) plotted on the FreeSurfer average brain. (**D**) Perception-related HFA modulation in AIC. HFA gains for detected versus missed trials across AIC electrodes, matched by load magnitude (participant means ± SE). The asterisk indicates significant difference (*P* < 0.01, signed-rank test). (**E**) Perception effects across the cortex. Normalized HFA gain differences (detected versus missed) for all regions. Asterisks indicate significant perception effects relative to unresponsive electrodes (*P < 0.05 and **P < 0.01). The inset shows the null effect in unresponsive sites (*n* = 1089). Related regions are grouped for clarity (see legend). Precentral includes primary motor, PMC, and SMA; parietal also includes postcentral and SMG; and frontal combines caudomedial and rostromedial areas. PMC, premotor cortex; SMG, supramarginal gyrus.

Participants reliably detected loads ≥5 cmH_2_O liter^−1^ s^−1^ (Fig. 2A), whereas lower loads were inconsistently detected near the perceptual threshold (Fig. 2B, yellow box; see fig. S3D for group data). This allowed classification of magnitude-matched load trials as detected or missed for neural comparison. In AIC electrodes (*n* = 36, eight participants; Fig. 2C), HFA responses were notably larger during detected versus missed trials (10.1% versus 5.0%; *P* = 0.002; Fig. 2D; individual participant data shown in fig. S5). Normalized gain ratios showed a 58% increase in AIC (Fig. 2E), with additional perception-related effects in orbitofrontal (43%) and fronto-motor regions (39%). Load-unresponsive electrodes (1089 of 1328) showed no such difference (*P* = 0.302; inset). Although inspiratory effort (iMP) was higher on detected trials, neural effects persisted after controlling for motor variability (fig. S6).

### Load response dynamics: AIC activity is transient, and frontal responses are sustained

**Fig. 3.**
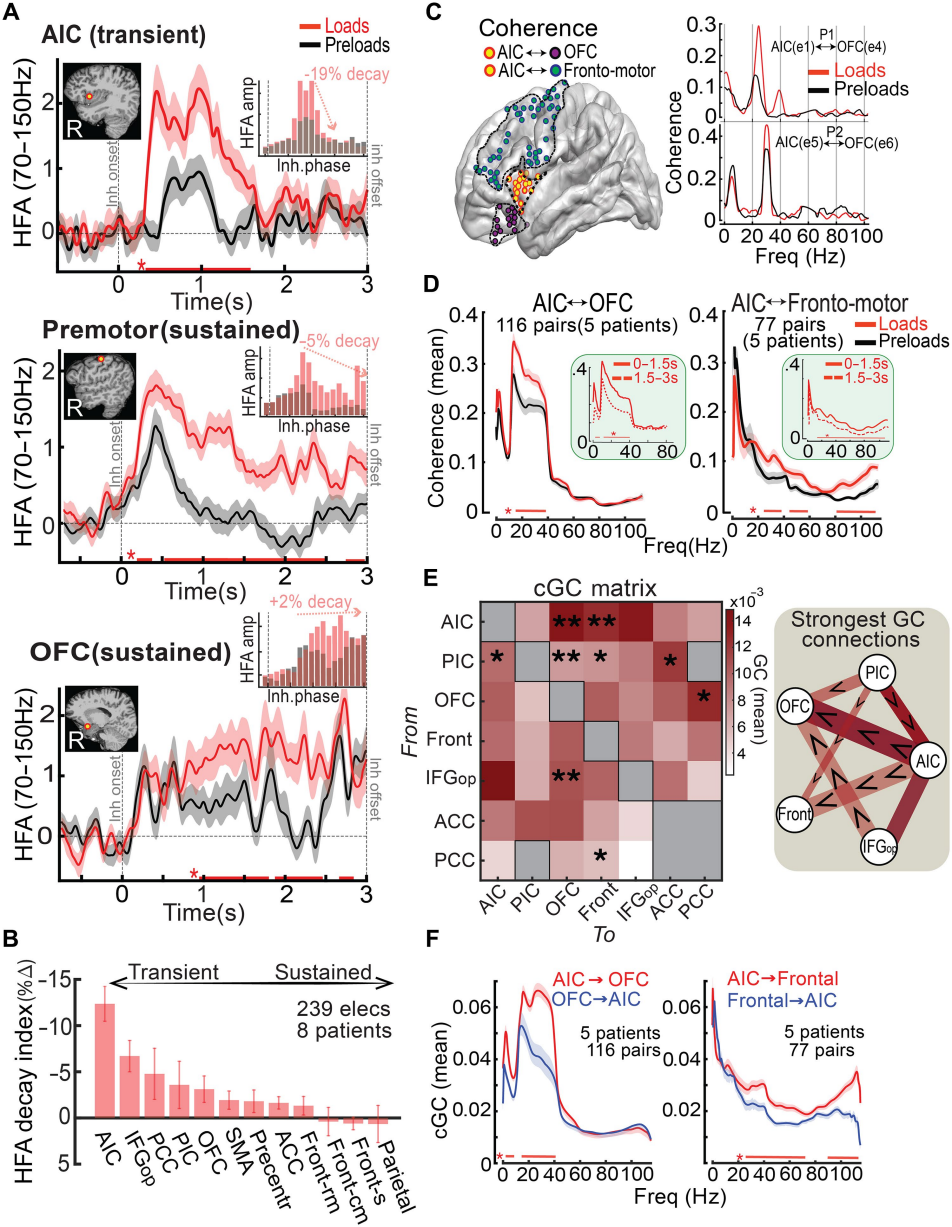
Temporal dynamics and network interactions associated with inspiratory load processing. (**A**) Transient versus sustained responses: HFA (70 to 150 Hz) evoked by inspiratory load in anterior insula, premotor cortex, and OFC from a representative participant (P5). Insets show HFA aligned to the inhalation phase, revealing a transient peak in AIC and sustained responses in frontal regions. (**B**) Population HFA decay index across regions (early versus late inhalation bins). AIC showed the steepest decline, consistent with transient engagement, whereas frontal regions exhibit sustained or minimal decay. (**C**) Electrode coverage and example coherence. Load-responsive electrodes in AIC, OFC, and fronto-motor regions plotted on the FreeSurfer average left brain for patients with simultaneous regional coverage (left). Example coherence spectra between AIC and OFC electrode pairs during load and preload conditions (0 to 3 s postinhalation; right). (**D**) Group-level coherence. Spectrally resolved coherence for AIC-OFC and AIC-fronto-motor pathways (precentral, rostromedial, and caudomedial). Insets show coherence during early (0 to 1.5 s) and late (1.5 to 3 s) phases of loaded inhalations. Sample sizes vary by region. (**E**) cGC matrix across load-responsive regions during loaded inhalations (0 to 3 s; 0 to 115 Hz). Darker colors indicate stronger GC magnitude (absolute influence); asterisks denote significant directional asymmetries. The schematic (right) summarizes dominant directional influences. (**F**) Spectrally resolved cGC. Directional GC spectra for AIC → OFC and AIC → fronto-motor pathways. Sample sizes vary by region.

To test whether the AIC initiates load detection, while frontal regions support downstream processing, we compared load-evoked HFA dynamics across regions. Example electrodes showed transient responses in the AIC and more sustained activity in premotor and orbitofrontal regions (Fig. 3A). To account for variability in inhalation duration (*31*), we normalized neural activity to the respiratory phase cycle (0° to 360°) and binned HFA accordingly (see insets and fig. S9). AIC responses were restricted to early-phase bins, whereas premotor and OFC regions remained active throughout the inhalation. Across participants, HFA declined by 12% across the AIC phase cycle but only 3% in the OFC (Fig. 3B), consistent with a temporal shift from early detection in AIC to prolonged processing in frontal cortices. Additional analyses of phase alignment, inhalation duration, and response latencies are provided in figs. S9 and S10.

These contrasting dynamics prompted us to test whether inter-regional coupling mirrored this temporal dissociation. We assessed coherence between AIC and both OFC and fronto-motor targets—regions selected on the basis of their strong and temporal distinct HFA responses (Fig. 3C). Coherence increased significantly during loaded versus preload trials (Fig. 3D), especially in the high β range (15 to 40 Hz) for AIC-OFC and across a broader band (20 to 115 Hz) for AIC-fronto-motor. Notably, coherence strength declined in the second half of inhalation (insets), consistent with a temporally restricted role for AIC signaling—initiating but not sustaining activity across downstream regions.

### AIC-to-OFC information flow predicts load detection

**Fig. 4.**
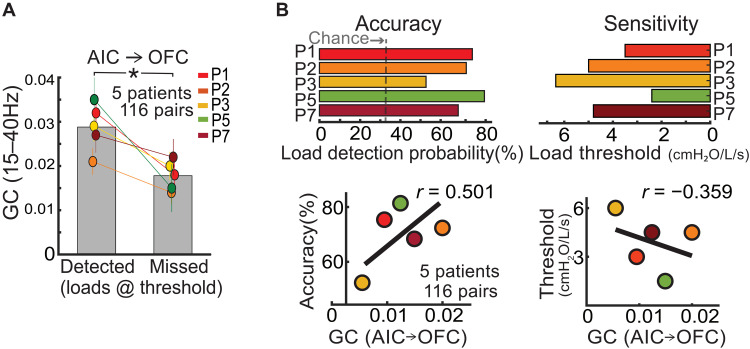
Directional AIC → OFC interactions are associated with load detection. (**A**) cGC from anterior insula to OFC in the β range (15 to 40 Hz) during loaded inhalations (0 to 3 s), comparing detected and missed trials (matched for load magnitude). Bars show group means across electrode pairs; colored dots and lines indicate individual participants. (**B**) Relationship between AIC → OFC cGC strength and behavioral performance. Left, stronger AIC → OFC cGC was associated with higher detection accuracy. Right, stronger cGC was associated with lower detection thresholds (greater sensitivity). Each point represents a participant average across electrode pairs.

While coherence analyses revealed strong, undirected coupling between AIC, OFC, and fronto-motor regions during loaded inhalations, they do not establish the direction of information flow. To address this, we applied spectral Granger causality (GC) (Fig. 3E). During loaded inhalations, GC analyses revealed robust AIC → OFC and AIC → fronto-motor interactions, with distinct spectral profiles: AIC → OFC connectivity peaked below 40 Hz, while AIC → fronto-motor influence spanned 20 to 115 Hz (Fig. 3F). These frequency-specific patterns may reflect different processing roles—interoceptive salience signaling versus motor compensation, respectively. Critically, only AIC → OFC connectivity increased during detected versus missed trials (*P* = 0.025; Fig. 4A) and tracked both detection accuracy and sensitivity (*r* = 0.501 and −0.359; Fig. 4B). Other pathways were weaker and not modulated by perception, underscoring the specificity of AIC → OFC signaling in supporting conscious load detection. Additional GC results are shown in fig. S8 and Supplementary Text (section S3). Notably, although GC magnitudes involving posterior insula (PIC) were smaller overall, PIC was the only region to show a consistent directed influence toward AIC during loaded inhalations (see Supplementary Text, section S7).

### AIC-lung coupling tracks load detection

**Fig. 5.**
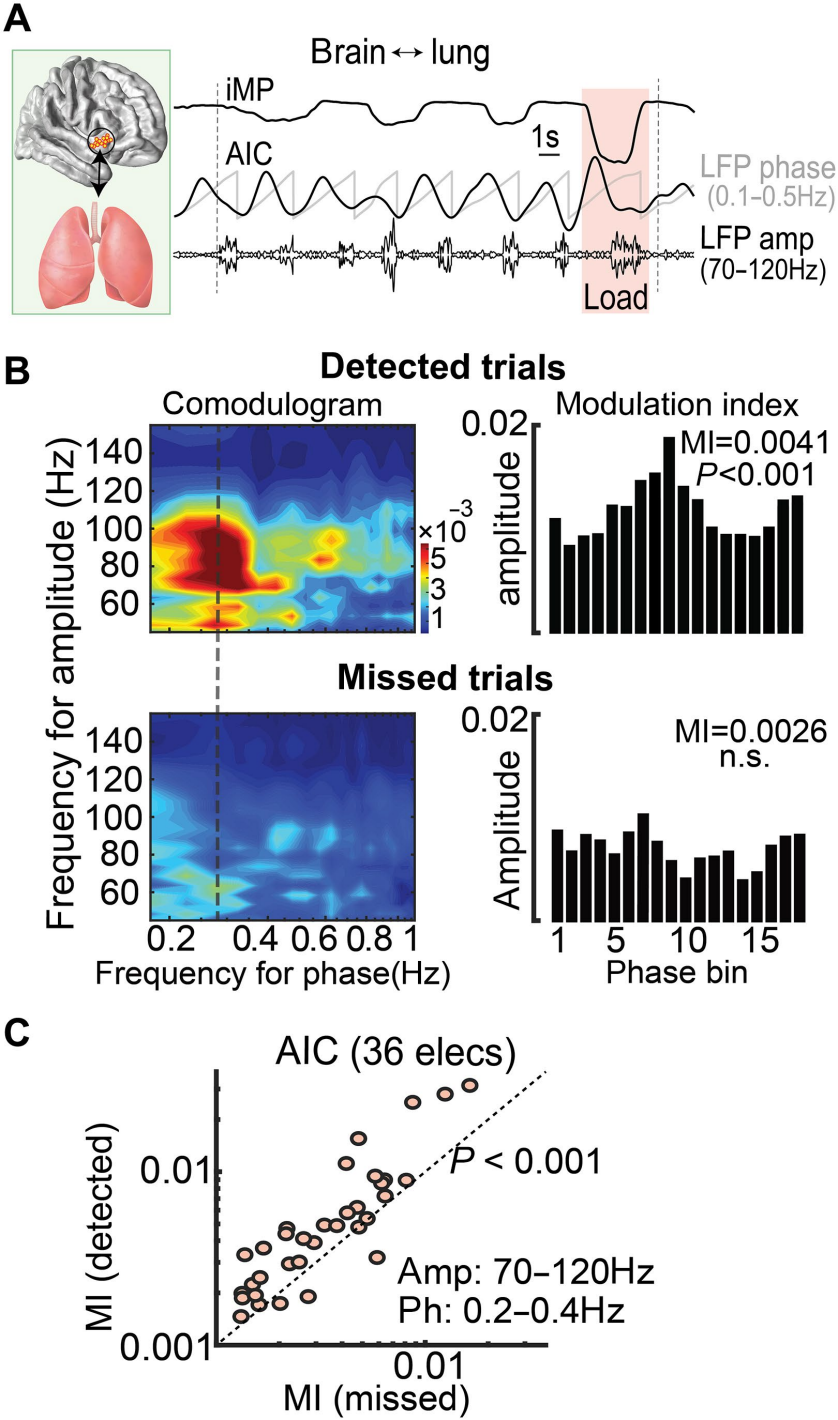
Respiratory phase-locking of AIC activity distinguishes detected from missed loads. (**A**) Schematic and example traces illustrating brain-lung coupling. Mouth pressure (iMP) and AIC LFPs from a representative electrode are shown during a trial. LFPs are filtered to extract the respiratory phase (0.1 to 0.5 Hz) and high-frequency amplitude (70 to 120 Hz). The shaded region marks the loaded breath. (**B**) PAC between the respiratory phase and AIC HFA. Left: Comodulograms showing PAC strength for detected (top) and missed (bottom) trials. Right: Mean high-frequency amplitude across respiratory phase bins and corresponding MIs. Significant PAC is present during detected trials but not during missed trials. (**C**) Comparison of PAC strength across AIC electrodes (*n* = 36). MIs are shown for detected versus missed trials (70- to 120-Hz amplitude; 0.2- to 0.4-Hz phase). PAC was significantly stronger during detected trials (*P* < 0.001).

Thus far, our analyses have focused on how inspiratory loads shape cortical activation and inter-regional communication. We next examined brain-lung coupling by asking how AIC activity aligns with the respiratory cycle during loaded breathing (Fig. 5A). Load-evoked HFA in AIC was modulated by the respiratory cycle in detected trials, whereas missed trials showed no such modulation (Fig. 5, B and C). Stronger respiratory-AIC coupling during detected trials was accompanied by larger local HFA gains (9.2% versus 5.0%; Fig. 2D and fig. S7A), linking AIC phase-coupling to successful load detection.

Notably, the absence of respiratory coupling in missed trials did not reflect a lack of cortical engagement. Even when low-magnitude loads fell below the perceptual threshold, AIC activity remained elevated, with load-evoked HFA increasing by ∼5% on average (Fig. 2D and fig. S7A), indicating that the AIC encodes respiratory constraints even without conscious awareness. These unconscious responses showed a distinct anatomical distribution, with relatively stronger gains outside the AIC—particularly in the anterior cingulate cortex (fig. S7B)—and were less sensitive to load magnitude than detected trials (fig. S7C). Together, these findings suggest that while basic load-related signals reach the cortex in both conditions, respiratory phase–locked AIC engagement distinguishes conscious detection from unconscious processing.

### Dissociable cortical encoding of inspiratory pressure and airflow

**Fig. 6.**
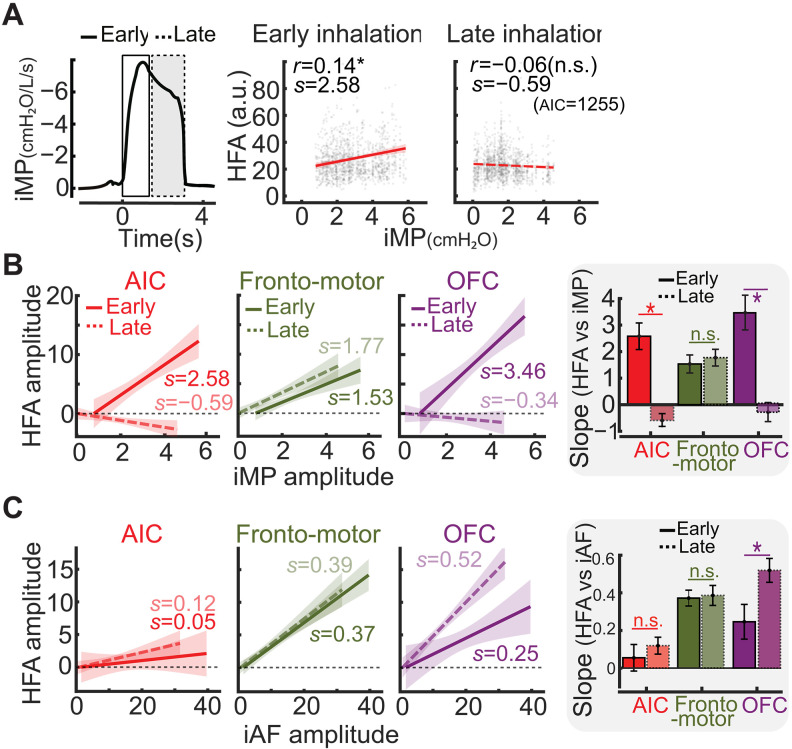
Regional encoding of inspiratory pressure and airflow across inhalation. (**A**) Example iMP trace segmented into early and late inhalation phases (left). Right: Trial-level relationships between HFA and iMP for all anterior insula electrodes, shown separately for early and late phases (dots: trials; lines: linear fits with Pearson *r* and slope). a.u., arbitrary units. (**B**) Same analysis for AIC, fronto-motor cortex, and OFC. AIC showed pressure-related HFA primarily during early inhalation, fronto-motor regions encoded pressure across both phases, and OFC showed stronger early-phase pressure sensitivity. Bar plots summarize group-level slopes (HFA versus iMP) across regions and phases (signed-rank tests, FDR-corrected). (**C**) Same layout as (B) for iAF. Fronto-motor regions encoded airflow across phases, AIC responses were weak, and OFC showed stronger late-phase airflow encoding. Bar plots summarize group-level slopes (HFA versus iAF); asterisks indicate significant effects.

To determine which respiratory features underlie brain-lung coupling, we correlated trial-level HFA with mouth pressure (iMP) and airflow (iAF) during early and late inhalation (Fig. 6 and fig. S11). AIC activity tracked iMP selectively during early inhalation, consistent with rapid mismatch detection of load onset. In contrast, fronto-motor regions showed sustained iMP-related modulation across both phases, and OFC also exhibited early-phase sensitivity to pressure. Slope comparisons confirmed significant regional and phase-specific differences in pressure encoding (Fig. 6B, inset).

Airflow correlations revealed a distinct spatiotemporal profile. Fronto-motor regions again showed strong, phase-invariant coupling, while AIC was only weakly modulated by iAF (Fig. 6C). In OFC, airflow encoding emerged later, becoming prominent during late inhalation. Together, these results dissociate cortical representations of inspiratory load components: The AIC preferentially signals early-phase pressure, consistent with rapid detection of mechanical mismatch, whereas OFC and premotor regions integrate both pressure and airflow over time, supporting sustained evaluation and motor compensation. This pattern reveals a functional division between early insular detection and downstream frontal integration and suggests a signal-specific organization in the forebrain that parallels brainstem tuning—such as airflow encoding in the preBötC (*1*) versus pressure/effort tracking in the periaqueductal gray (PAG) (*8*).

### Perceived load intensity modulates responses in the sensory cortex

**Fig. 7.**
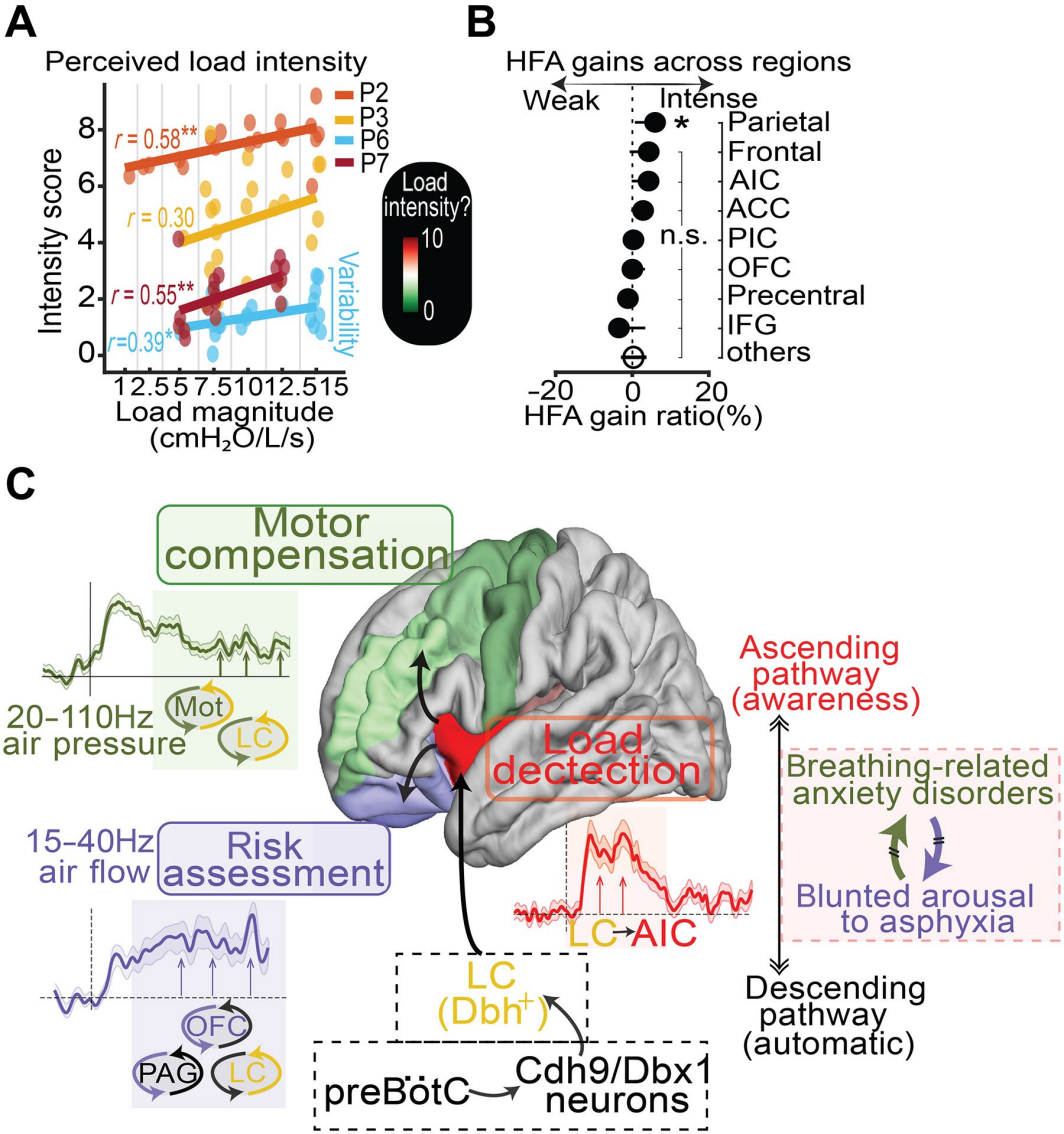
Load intensity encoding and a proposed cortical-brainstem model of respiratory perception. (**A**) Perceived intensity. Higher magnitude loads were rated as more intense on a VAS (*n* = 4 participants). Lines show within-participant relationships between load magnitude and perceived intensity. (**B**) Neural correlates of perceived intensity. HFA in the parietal cortex was greater for trials rated as intense versus weak after matching trials for load magnitude. Effects are shown across parietal electrodes defined by the Desikan-Killiany atlas, including postcentral and supramarginal regions. (**C**) Proposed model. Inspiratory loads engage ascending brainstem-cortical pathways supporting awareness and descending pathways supporting automatic compensation. Corollary discharge from preBötC Cdh9/Dbx1^+^ neurons may reach the LC and anterior insula to support mismatch detection between expected and actual airflow. AIC signals are routed to OFC for appraisal or risk evaluation and to the premotor cortex for motor adjustment. OFC may engage descending projections to LC, PAG, and olfactory bulb (OB), forming recurrent loops that sustain appraisal during loaded breathing. Late-phase OFC airflow encoding and sustained fronto-motor pressure encoding are consistent with parallel perceptual and compensatory processes. Dashed elements indicate hypothesized pathways and affected populations.

Participants rated perceived load intensity after each trial using a 0-to-10 visual analog scale (VAS; Fig. 1B). Ratings correlated with load magnitude in each of the four participants with complete data; when pooled across all trials, the correlation was *r* = 0.555 and *P* = 0.017 (Fig. 7A). Notably, identical loads (e.g., 15 cmH_2_O liter^−1^ s^−1^) were sometimes rated as weak and other times as intense. Neural activity in the parietal cortex, AIC, and frontal cortex was modestly higher during trials rated as more intense despite matched load magnitudes. This effect reached significance only in the parietal cortex (*P* = 0.045; Fig. 7B), suggesting a role in encoding perceived load intensity.

### Load magnitude effects

Neural responses scaled with load magnitude across regions but differed in growth profiles (Fig. 2, A and B; see also fig. S12). In the AIC, responses increased with lower loads (≤10 cmH_2_O liter^−1^ s^−1^) but plateaued or declined at higher loads. In contrast, OFC responses showed a more linear increase (fig. S12, C and D). A saturation index (SI) confirmed this pattern: AIC and pars opercularis exhibited early saturation, while other regions maintained more consistent gains (fig. S12E). Additional magnitude effects and individual participant data are presented in fig. S12 and Supplementary Text.

### Control analyses ruled out nonspecific effects of breath order, timing, or muscle artifacts

To ensure that load-related HFA increases were not driven by confounds such as breath position, motor readiness, or anticipation, we performed several control analyses. First, instead of contrasting loaded breaths with the immediately preceding preload, we compared loaded inhalations to no-load breaths matched for breath position from adjacent trials. This yielded nearly identical electrode coverage (fig. S13B) and effect sizes, with no significant difference in HFA gain (Wilcoxon signed-rank test, *P* = 0.11, *z* = 1.61; fig. S13C).

Second, preinspiratory HFA (−1 to 0 s) remained stable across successive breaths (fig. S13D), indicating no drift in baseline cortical excitability. Friedman tests showed no significant breath-order effects within any region (all *P* > 0.06; e.g., AIC: χ^2^ = 2.55, *P* = 0.47; OFC: χ^2^ = 0.75, *P* = 0.86) nor when averaging across regions (χ^2^ = 0.75, *P* = 0.86).

Third, cue-to-inhalation latency did not differ across breaths, indicating stable behavioral timing throughout the task [repeated-measures analysis of variance (ANOVA), *F*_3,21_ = 1.05, *P* = 0.39]. Consistently, within-participant Friedman tests also revealed no breath-wise effects (all *P* > 0.21).

Last, to address potential contamination of broadband HFA by orofacial or neck muscle activity, we reanalyzed the data using a narrower γ band (35 to 75 Hz). The key load–related and perceptual effects persisted with similar spatial distribution and magnitude (fig. S14), supporting a cortical rather than muscular origin.

## DISCUSSION

We provide direct intracranial electrophysiological (iEEG) evidence for two distinct cortical populations involved in human breathing interoception (respiroception). One, in the AIC, drives the conscious detection of subtle respiratory anomalies. This region exhibits transient, load-evoked activity consistent with early mismatch detection between expected and actual inspiratory dynamics and relays sensory signals to downstream cortical targets (*16*). AIC activity alone outperformed subjective reports in identifying near-threshold loads. Notably, information flow from AIC to OFC improved load detection and scaled with both detection accuracy and perceptual sensitivity (Fig. 4), pointing to a cortical mechanism underlying interindividual variability in breathing awareness.

A second population, spanning the fronto-motor cortex and OFC, showed sustained load-evoked activity with distinct tuning across respiratory phases (Figs. 3A and 6). Premotor and rostromedial regions encoded both mouth pressure and airflow throughout the entire inspiration cycle, consistent with compensatory motor control and with prior EEG findings linking these regions to breathing effort (*17*, *32*, *33*). In contrast, the OFC tracked pressure early and airflow later, suggesting a shift from initial load appraisal to downstream perceptual evaluation. These dynamics support a model in which the AIC detects early deviations in expected inspiratory effort, the premotor cortex mediates motor compensation, and OFC integrates the sensory and behavioral significance of the load. This cortical dissociation between inspiratory pressure and airflow echoes the functional segregation seen in brainstem centers, where regions such as the PAG encode effort and load perception (*8*, *34*), while the preBötC and nucleus tractus solitarius (NTS) track airflow dynamics and timing (*1*, *5*).

We propose that the AIC acts as a cortical gateway for ascending respiratory signals. These inputs may travel via the olfactory bulb (*35*, *36*)—unlikely here due to nasal occlusion—a somatosensory route (*37*), or most plausibly, a corollary discharge pathway from brainstem centers (*38*, *39*). This latter route likely involves excitatory input from inspiratory-active Cdh9/Dbx1^+^ neurons in the preBötC (*9*), which may be preferentially engaged during consciously detected inspiratory loads (Fig. 4E). The resulting corollary discharge could enhance mismatch detection in the AIC by flagging discrepancies between predicted and actual inspiratory efforts, aligning with previous evidence implicating the AIC in error monitoring (*40*–*42*) and interoception (*15*, *43*).

Beyond initiating detection, routing of AIC signals to the OFC appears essential for successful perception and higher-order evaluation of respiratory stimuli (Figs. 3, E and F, and 4). The sustained OFC response likely reflects integration of stimulus salience or threat, maintained through recurrent interactions with brainstem centers throughout the loaded inhalation. Previous work has implicated the OFC in encoding behavioral relevance (*44*), including in respiratory contexts (*45*), and has shown its direct projections to PAG subregions involved in respiratory control (*34*, *46*). Through these pathways, the OFC may initiate descending loops that sustain cortical appraisal and modulate ventilation by recruiting noradrenergic [locus coeruleus (LC)] and respiratory rhythm–generating (preBötC) circuits. Disruption of this AIC-OFC-brainstem network may explain not only missed detections in the IRDT task but also blunted arousal responses in life-threatening conditions such as opioid-induced respiratory suppression, sudden infant death syndrome, COVID-19 pneumonia, and severe asthma (Fig. 7C) (*18*–*22*).

Premotor and rostromedial frontal regions also showed robust load-evoked responses and received input from the AIC, notably at higher frequencies (20 to 110 Hz) than the AIC-OFC pathway (15 to 40 Hz). This spectral dissociation suggests distinct functional roles: Lower-frequency AIC-OFC interactions may support sustained perceptual appraisal, while higher-frequency AIC-motor coupling likely reflects rapid motor adjustments. Prior functional magnetic resonance imaging (MRI) studies demonstrate involvement of both insular and premotor cortices in response to inspiratory mechanical loads (*15*, *47*, *48*), and EEG studies show early supplementary motor area (SMA) activation followed by broader frontal engagement (*17*, *32*). Our iEEG data clarify this spatiotemporal cascade, revealing strong responses in medial precentral gyrus extending into the caudomedial and rostromedial frontal cortex. Notably, unlike AIC-OFC pathways, AIC-motor projections did not correlate with detection performance—suggesting that these circuits support ventilatory compensation rather than conscious perception. This dissociation may help explain motor-perceptual decoupling in anxiety-related breathing disorders, such as hyperventilation without awareness (*23*, *24*).

Our findings define a cortical AIC-OFC-motor circuit that links respiratory perception to action, forming a core substrate for interoceptive awareness and a potential target for restoring adaptive breathing control. While this circuit was delineated using transient inspiratory loads, other respiratory challenges, including those associated with emotional or metabolic stress, may engage this network (*49*) or recruit additional regions, such as the amygdala (*12*, *50*). Together with recent neuroimaging work dissociating insular, prefrontal, and somatosensory contributions to respiratory perception (*48*), our findings align with hierarchical interoceptive models (*51*) in which early sensory representations are transformed into detection and appraisal signals via anterior insula-frontal interactions. By mapping these core pathways, we provide a foundation for future studies to probe how emotional and cognitive functions shape the subjective experience of breathing and its dysregulation in disease.

## MATERIALS AND METHODS

### Participants

We recorded data from neurosurgical patients with drug-resistant epilepsy undergoing iEEG monitoring at North Shore University Hospital (New York). This study was approved by the Feinstein Institutes for Medical Research Institutional Review Board (protocol nos. 07-125 and 25-0306). All participants provided written informed consent in accordance with the Declaration of Helsinki. No compensation was provided.

Eleven patients (five females; age: 19 to 54 years; mean: 34.2) completed the IRDT (table S1). None had a history of smoking or cardiorespiratory disease. As part of postoperative care, all were on low-dose antiepileptics and/or analgesics; testing was timed during the wear-off period to minimize drug effects. Pulmonary function was assessed before the IRDT using a hand-held spirometer, confirming normal lung function in all participants (FEV_1_, FVC ≥ 80% predicted; FEV_1_/FVC ≥ 0.70, adjusted for height) (see table S2). The body mass index ranged from 19.3 to 29.4 kg/m^2^. During testing, patients were seated in a reclining hospital bed with support for the back, arms, neck, and head to minimize motion artifacts.

### Breathing circuit

A silicon mouthpiece (with nose clip) ensured accurate respiratory measurements, avoiding air leaks common with face masks (*30*, *52*). Inspiratory flow was measured using a pneumotachograph (3700 series, Hans Rudolph; linear range: 0 to 160 liters/min) coupled to a 5 cmH_2_O differential pressure transducer (DP45-18, Validyne). The setup incorporated a two-way nonrebreathing valve (2700 series, Hans Rudolph). Inspiratory airway pressure was recorded via a lateral mouthpiece port connected to a separate transducer (DP15-32, Validyne; 0 to 140 cmH_2_O range). The inspiratory port was manually switched to a threshold-loading device just before target inhalation (e.g., on breath no. 3 or 4 in each trial). Manual switching was performed silently from behind the participant, with blinding and cue minimization procedures detailed in Supplementary Text (section S1). Expiration was unrestricted and not measured. Participants wore noise-canceling earplugs to eliminate ambient and self-generated breathing sounds. All tubing was securely fixed to prevent displacement or contact. Respiratory signals were sampled at 100 Hz using a SmartLab system (Hans Rudolph). Peak and average inspiratory flow and pressure values were extracted from a 0- to 3-s window after inhalation onset.

### Task and stimuli

Stimuli were presented at bedside using the Psychophysics Toolbox version 3 in MATLAB (MathWorks). Each trial began after the participant fitted the mouthpiece and nose clip and pressed a key to display a small fixation ring on the screen (Fig. 1B). Participants fixated the ring and breathed at their natural pace and volume for 5 s (−7:−2 s). The ring then turned green (−2:0 s), signaling them to prepare to inhale. Next, the ring gradually expanded (0 to 3 s) and contracted (3 to 6 s); participants synchronized their inhalations/exhalations to match this rhythm (paced breathing: 3-s inhalation and 3-s exhalation) for four consecutive breaths. A 500-ms pause after each exhalation mimicked natural breathing.

In catch trials, no resistance was applied. In loaded trials, an inspiratory flow resistive load was randomly introduced on the third or fourth breath. Participants could not see whether (or when) a load was delivered. After the fourth inhalation, they removed the mouthpiece and answered: Which breath was harder? (3 versus 4 versus all-equal; Fig. 1B). If they reported a harder breath, they rated its intensity on a 0-to-10 VAS, where 0 indicated a barely noticeable or very light load and 10 indicated a very strong or intense load. Each participant completed at least 38 trials (range: 38 to 59), receiving load magnitudes both above and below their perceptual threshold. Load levels were 1, 2.5, 5, 7.5, 10, 12.5, and 15 cmH_2_O liter^−1^ s^−1^, each presented at least four times. Loads were calibrated beforehand for linearity. Control trials without loads measured false alarms.

Participants were instructed to breathe in sync with the ring as naturally (i.e., without increasing tidal volume) and as stably (uniform across breaths) as possible (see Supplementary Text). This controlled respiratory rate and tidal volume, minimizing differences across participants and conditions. Because loads were relatively mild, applied to only one inspiration per trial, and no participants reported breathlessness during initial training (zero breathlessness scores), we did not collect breathlessness ratings during the main task to save time. Similar tasks have recently been used to assess respiratory sensitivity in behavioral (*28*) and neuroimaging studies (*15*).

### iEEG data acquisition

Patients were implanted with either depth electrodes (2- or 1.3-mm platinum cylinders; 4.4- or 2.2-mm center-to-center spacing; 0.8-mm diameter) or subdural strips/grids (2- or 3-mm platinum discs; 4- or 10-mm spacing; PMT Corporation), with placement determined by clinical needs. iEEG was recorded at 1.5 kHz (Tucker-Davis Technologies) or 1 kHz (XLTEK Quantum, Natus Medical) and referenced online to an electrode beneath the skull. Transistor-transistor logic pulses sent by the stimulus software were simultaneously recorded with the iEEG to align neural and respiratory data.

### Electrode localization

Electrode localization and visualization were performed in MATLAB using the iELVis toolbox (*53*). Before implantation, each participant underwent a T1-weighted 1-mm isometric structural MRI scan on a Siemens 3T MRI scanner. After implantation, a computed tomography (CT) scan and T1-weighted MRI were acquired and co-registered to the preoperative MRI using FSL’s BET (*54*) and FLIRT algorithms (*55*). This minimized localization errors from brain shift and allowed CT to overlay precisely on the preoperative MRI. Electrode contacts were then semimanually identified on the co-registered CT in BioImageSuite (*56*). Volumetric data from the preoperative T1 were processed with FreeSurfer (“recon-all,” version 6.0.0) (*57*). For surface visualization, depth electrodes were snapped to the nearest point on the FreeSurfer pial surface.

### Data preprocessing

We selected 11 neurosurgical patients on the basis of intracranial electrode coverage of interoceptive and respiratory-related cortical regions, with a total of 1768 implanted sites. Three were later excluded because of task noncompliance or excessive epileptiform activity (table S1), yielding 8 participants (6 with depth electrodes and 2 with both depth and grid arrays). Contacts showing abnormal epileptiform activity, involvement in seizure onset zones (per clinical evaluation), or placement outside brain tissue were excluded. We also discarded electrodes fully within white matter, defined by proximal tissue density (PTD < −0.9) (*58*). iEEG time series were visually inspected for signal quality, and electrodes with systematic artifacts or interictal spikes [detected by an automated spike detector (*59*)] were removed. This process yielded 1328 electrodes for analysis (Fig. 1E and fig. S1).

iEEG data were notch filtered at 60, 120, and 180 Hz, bandpass filtered from 0.01 to 200 Hz, downsampled to 500 Hz, and re-referenced to the average across electrodes. Data were segmented into 45-s epochs containing all task events within a trial. iEEG and respiratory signals were aligned, and inhalation onsets were detected when iAF exceeded 0.2 liters/min or iMP dropped below −0.2 cmH_2_O, accounting for load-related delays. Each inhalation was epoched from −6 to +6 s relative to onset. Epochs with transient artifacts were rejected by visual inspection before comparing preloaded and loaded inhalations.

### HFA analysis and selection of load-responsive electrodes

HFA (70 to 150 Hz) was extracted as a neural response measure because of its correlation with local multiunit firing (*60*, *61*). HFA was computed by bandpass-filtering the signal into 10-Hz bands from 70 to 150 Hz (fourth-order Butterworth, “filtfilt”), applying a Hilbert transform to extract the instantaneous amplitude, normalizing by the time-series mean, averaging across bands, and squaring to obtain power.

Electrodes were classified as load-responsive (Fig. 1E and fig. S1) if their HFA was significantly higher during loaded versus preloaded inhalations within the 0- to 3-s postinhalation window (*P* < 0.05, sign-rank test, >2%Δ; range: 38 to 59 trials per participant; seven load magnitudes). HFA during preloaded inhalations (0 to 3 s) served as the baseline, and HFA gain was computed as

$$\text{HFA}\;\text{gain}=\left({\text{HFA}}_{\text{load}}-{\text{HFA}}_{\text{preload}}\right)/{\text{HFA}}_{\text{preload}}\times 100$$

HFA gains for all electrodes within a brain region were then averaged to yield one overall HFA gain per region with *P* values corrected via false discovery rate (FDR) (*62*).

### Perception-dependent load effects

For each participant, we identified trials where loads were applied but not detected (missed) and matched them to the nearest detected trials of the same load magnitude. HFA gains (%ΔHFA detected versus missed, 0 to 3 s postinhalation) were averaged within each electrode and brain region. These values were compared across participants using two-sided Wilcoxon signed-rank tests (Fig. 2D). HFA gain ratios were calculated for each electrode as

$$\begin{array}{c} \text{HFAgainratio}=\left[\left(\text{HFAgain\_detected}-\text{HFAgain}\_\text{missed}\right)/\right.\\ \left.\left(\text{HFAgain}\_\text{detected}+\text{HFAgain}\_\text{missed}\right)\right]\times 100 \end{array}$$

HFA gain ratios were then averaged within each region (Fig. 2E) and compared against those from load-unresponsive sites using Wilcoxon rank-sum tests, with *P* values corrected for multiple comparisons via the original FDR approach (*62*). For missed trials alone (fig. S7), HFA gains (%ΔHFA load versus preload) were averaged across load-responsive electrodes within each region and compared to unresponsive regions. Load dependence was assessed by comparing HFA gains across load magnitudes within each region (Fig. 2B and fig. S7C).

### Transient versus sustained responses

To capture transient versus sustained load effects, we first accounted for variability in inhalation duration (fig. S9, A and B) (*31*). We extracted the inhalation phase using a Hilbert transform on an inspiratory efficacy signal (iAF/iMP), which provided a stable phase estimate (Fig. 3A, insets, and fig. S9). Phases from −π (onset) to +π (offset) were binned into 30° segments (12 bins). Inspiratory and HFA amplitudes were averaged per bin, producing phase-amplitude distributions for loaded (red) and preloaded (black) trials (fig. S9, C and D). Bins were compared via Wilcoxon rank-sum tests, and results were aggregated across electrodes within each brain region (fig. S9F).

To quantify suppression across the respiratory cycle, we computed an HFA decay index (Fig. 3B). Inspiratory efficacy signals were segmented into active, hold, and passive phases using a slope-based method [Breathmetrics toolbox (*63*)]. Mean HFA was extracted for each phase and then fit with a linear regression. Negative slopes indicated HFA decay from early to late phases; positive slopes indicated increasing trends. These indices were averaged per electrode and then across electrodes within each region. Additional methodological details are provided in Supplementary Text (section S4).

### Spectral coherence

To quantify undirected functional coupling between regions during inspiratory processing, we computed magnitude-squared spectral coherence between electrode pairs (Fig. 3, C and D). Coherence was estimated using the same preprocessing, multitaper spectral estimation parameters (time-bandwidth product NW = 2, 4 tapers), time windows (0 to 3 s post–inhalation onset), and frequency range (0 to 115 Hz) as the GC analyses, ensuring direct comparability across measures. Coherence spectra were averaged across load-responsive electrode pairs within each region pair and contrasted between loaded and preload conditions, as well as between early (0 to 1.5 s) and late (1.5 to 3 s) inhalation windows.

### Granger causality

To assess the directionality of functional interactions underlying respiratory perception, we applied conditional GC (cGC), which controls for shared inputs (*64*, *65*). Analyses focused on regions showing robust load responses: AIC, OFC, pars opercularis, frontal cortex (precentral, caudomedial, and rostromedial combined), PIC, and anterior and posterior cingulate cortices (ACC and PCC, respectively). GC was computed on load-responsive electrode pairs within each participant using data from loaded inhalations (0 to 3 s post–inhalation onset, 0- to 115-Hz range).

Local field potentials were demeaned and bipolarized by subtracting the nearest white matter signal to minimize volume conduction. Directional interactions were quantified using a nonparametric, frequency-domain GC approach on the basis of Wilson’s spectral factorization (*66*), implemented with publicly available code. Spectral estimates were computed using multitaper methods (time-bandwidth product NW = 2, 4 tapers) on 3-s windows sampled at 500 Hz. Zero padding was applied to obtain a frequency resolution of 0.33 Hz. Because this approach does not rely on autoregressive modeling, model order selection was not required. Pairwise-conditional GC was computed across the full 0- to 115-Hz range in both forward and reverse directions. GC values were averaged within each region pair and summarized in matrix form (Fig. 3E). Directional asymmetries were assessed by comparing forward versus reverse GC using two-sided Wilcoxon signed-rank tests, with FDR correction for multiple comparisons (*62*).

Frequency-resolved GC profiles were averaged across electrode pairs within each connection and compared using Wilcoxon signed-rank tests (Fig. 3F). To assess perceptual relevance, GC was further compared between detected and missed trials matched for load magnitude (Fig. 4A). Last, detection accuracy and perceptual thresholds were correlated with GC strength using Pearson correlations (Fig. 4B).

### Phase-amplitude coupling

We assessed how the phase of slow respiratory oscillations modulated high-frequency amplitude in the AIC (Fig. 5A). Mouth pressure (iMP) was bandpass filtered at 0.1 to 0.5 Hz to extract the respiratory rhythm, and a Hilbert transform was applied to compute the instantaneous phase. HFA (70 to 150 Hz) was extracted as described above.

We quantified phase-amplitude coupling (PAC) using the modulation index (MI) on the basis of the Kullback-Leibler method (*67*). Briefly, HFA amplitudes were sorted into 18 phase bins across the respiratory cycle, and deviations from a uniform distribution were measured as MI. Comodulograms were constructed by varying phase frequencies (0.1 to 1.2 Hz) and amplitude frequencies (70 to 150 Hz) to identify peak coupling (Fig. 5B).

For group analysis (Fig. 5C), we focused on the phase at 0.2 to 0.4 Hz and amplitude at 70 to 120 Hz, matching typical inhalation rates and broadband HFA. MIs were computed separately for detected and missed trials (matched for load magnitude) and compared within each AIC electrode using paired Wilcoxon signed-rank tests across the pooled set of electrodes (*n* = 36 from eight participants).

### Correlation analysis between HFA and iMP or iAF

To assess how cortical activity encodes mouth pressure or airflow during inspiratory load, we correlated trial-level HFA (70 to 150 Hz) with iMP or iAF amplitudes during early (active) and late (passive) inhalation phases (Fig. 6). For each electrode, we extracted per-trial HFA and iMP/iAF amplitudes and computed Pearson correlations and linear regression slopes within predefined regions of interest. Correlations and slopes were calculated separately for each phase and region. We assessed statistical significance using Pearson correlation (for within-region effects) and signed-rank tests on slope distributions (for between-region comparisons). To directly test for slope differences between regions, we fit a combined linear model with an interaction term (region × signal amplitude), treating region as a categorical predictor, using MATLAB’s fitlm function. FDR correction was applied across all tests. For visualization, regression overlays were aligned such that HFA was set to zero at the minimum iMP/iAF value, enabling direct slope comparisons. Summary panels display statistical differences in slope strength across regions and inhalation phases (see fig. S11 for details).

### Load magnitude responses and saturation profiles

HFA gains were computed as described above but separately for each load magnitude (1, 2.5, 5, 7.5, 10, 12.5, and 15 cmH_2_O liter^−1^ s^−1^), yielding values such as HFAgain-5 for 5 cmH_2_O liter^−1^ s^−1^. Each participant completed at least 38 trials (mean: 42; range: 38 to 59), with each load presented at least four times. Gains across magnitudes were fitted with weighted polynomial regression, assigning weights on the basis of the standard deviation (SD) at each magnitude, to generate a load-response function for each electrode (fig. S12).

To quantify each electrode’s dynamic response range, we calculated an SI from the slopes between consecutive data points of the load-response function, normalized by the total absolute slope (fig. S12E). Negative slopes at higher load magnitudes were weighted more heavily to emphasize flattening. SI ranges from −1 (purely increasing response) to +1 (strong saturation), with values near 0 indicating balanced changes. In particular

$$\text{SI}=\left[\sum w_{i}\times |\Delta y_{i}/\Delta x_{i}|\times \text{sign}\left(\Delta y_{i}\right)\right]/\left(\sum |\Delta y_{i}/\Delta x_{i}|\right)$$

where Δ*y_i_*/Δ*x_i_* = (*y*_*i*+1_ − *y_i_*)/(*x*_*i*+1_ − *x_i_*) is the slope between consecutive points, and weights were defined as *w_i_* = *x_i_*/max(*x*). SIs were averaged across electrodes within each brain region to yield one overall SI per region.

### Load response latencies

The onset latency of HFA differences between preloaded and loaded inhalations was estimated using the phase-binned method described above (here with 24 bins for a higher resolution) and then mapped back to real time on the basis of single-trial bin timing. A complementary time-domain approach, using a sliding sign-rank test (*P* < 0.05), produced similar results, confirming the robustness of these latency estimates. Only brain regions with consistent latency estimates across both methods and at least five electrodes were included in the summary plot (fig. S10C).

### Control analyses for breath order, arousal, and muscle artifacts

To rule out confounding effects from breath order, anticipatory arousal, or muscle-related artifacts, we conducted four control analyses: (i) Loaded breaths were compared to matched-position no-load trials (e.g., breath 3 versus breath 3 across trials) to ensure that observed effects were not driven by breath position or trial structure. (ii) Preinspiratory HFA (−1 to 0 s) was tested for stability across the four-breath sequence using Friedman tests, both within each region (with subjects as repeated measures) and across regions. (iii) Cue-to-inhalation latencies (from ring expansion to inspiratory onset) were measured across breaths to assess potential changes in motor readiness or timing. (iv) To address potential contamination of broadband HFA (70 to 150 Hz) by perioral muscle activity, we reanalyzed all data using a narrower γ band (35 to 75 Hz). Together, these analyses confirmed consistent neural baselines and timing across breaths and showed that key load–related and perceptual HFA effects persisted even under stricter frequency constraints. These controls support a cortical origin for the observed HFA increases, rather than artifacts from arousal, expectancy, or electromyography (see figs. S13 and S14).

### Local activation versus inter-regional communication

HFA (70 to 150 Hz) was used to index local population-level cortical activation, which is relatively insensitive to volume conduction. Inter-regional interactions were assessed using coherence and cGC, which quantify frequency-specific coupling and directed influences between regions. Because long-range cortical interactions are typically expressed at lower frequencies, coherence and Granger analyses were computed across the full spectral range with emphasis placed on frequency bands showing the strongest load or task-related effects.

### Statistical analysis

Research staff was blinded to the experimental condition (load magnitude: 0, 1, 2.5, 5, 7.5, 10, 12.5, or 15 cmH_2_O liter^−1^ s^−1^) during data collection and to the detection outcome (detected versus missed) during data preprocessing and analysis. All recorded trials from each participant were included unless excluded for technical artifacts (e.g., amplifier saturation, electrode disconnection, and epileptiform discharge) or behavioral noncompliance (e.g., premature trial termination). No trials were excluded on the basis of the neural response. Participants were not randomized, as all underwent the same experimental protocol.

Sample size was determined by the availability of patients who met three criteria: (i) clinically implanted intracranial electrodes in potentially relevant interoceptive and sensorimotor regions, (ii) ability to perform the detection task (detecting at least the largest loads), and (iii) absence of excessive interictal spiking in those regions. This cohort size is consistent with prior intracranial studies of respiratory interoception (*3*, *39*, *50*) and provides sufficient statistical power to detect large within-subject effects observed in similar designs.

When the normality assumption (Shapiro-Wilk test) was met, paired Student’s *t* tests were used to compare two within-subject conditions, one-way repeated-measures ANOVA to compare more than two conditions, and two-way repeated-measures ANOVA to adjust for additional within-subject factors (e.g., respiratory phase). Nonparametric counterparts (Wilcoxon signed-rank test and Friedman test) or the rank-sum test for unequal sample sizes was applied when the normality assumption was violated. Pearson correlation coefficients were used to assess linear relationships between variables; significance values were computed using two-tailed tests. Effect sizes were expressed as the percent change from the baseline (preload), unless otherwise noted. For cross-electrode and cross-participant comparisons, responses were normalized by computing ratios relative to the baseline condition. FDR correction was applied to account for multiple comparisons, as specified in the figure legends. For a supplementary analysis (fig. S12), gains across load magnitudes were fitted with weighted polynomial regression to generate a load-response function for each electrode.

## Supplementary Materials

### This PDF file includes:

Supplementary Text Figs. S1 to S15 Tables S1 and S2 References
