# Supplementary material for: Insular routing to orbitofrontal cortex enables breathing awareness
Source: Sci Adv. 2026 Jul 29;12(31):eaeb3326. doi: 10.1126/sciadv.aeb3326 (PMC13418533; doi:10.1126/sciadv.aeb3326)
Supplement: Supplementary file 1 — Supplementary Text Figs. S1 to S15 Tables S1 and S2 References [file sciadv.aeb3326_sm.pdf]

Science Advances
AAAS

Supplementary Materials for

Insular routing to orbitofrontal cortex enables breathing awareness

Joshua Y. Assi *et al.*

Corresponding author: José L. Herrero, jherreroru@northwell.edu

Sci. Adv. **12**, eaeb3326 (2026)
DOI: 10.1126/sciadv.aeb3326

This PDF file includes:

Supplementary Text
Figs. S1 to S15
Tables S1 and S2
References

Supplementary Text

1. Advantages of the Inspiratory Resistance Detection Task

Previous intracranial studies examined respiration–neural correlations but did not directly probe the respiratory system (3, 39, 50). Our inspiratory resistance detection task (IRDT) introduces calibrated inspiratory flow-resistive loads to elicit measurable effort and directly assess respiratory interoception. The IRDT was inspired by prior respiratory interoception paradigms, including the Respiratory Resistance Sensitivity Task (RRST; 28) and respiratory learning protocols (15). Unlike the RRST, which uses a two-interval forced-choice (2IFC) design and computes d′ or detection thresholds under criterion-free assumptions, our task uses a three-alternative forced-choice format ("Which breath was harder?") across four paced breaths, with resistance applied on breath 3, 4, or not at all. This design stabilizes tidal volume over four breaths, reduces predictability, and introduces catch trials - features that enhance perceptual sensitivity in a more natural context, while precluding direct d′ estimation. For physiological analyses, loaded breaths were compared to the immediately preceding preload, as in previous clinical and cognitive loading studies (15, 28, 47). Control analyses using matched breath positions (e.g., breath #3 across trials) produced similar results (see Fig. S13 and section 8).

To minimize non-respiratory cues and maintain experimenter blinding, the load valve was triggered manually from behind the participant using pre-randomized instructions (e.g., "load on breath 3" or "breath 4"), but the experimenter remained blinded to load magnitude —including catch trials (0 cmH_2O). The valve mechanism used a low-friction sliding PVC switch (Hans Rudolph Three-Way T-Shape Manual Stopcock-Type, model SDP) integrated into fixed tubing, enabling silent, smooth transitions with no visible movement. Tubing was secured and positioned outside the participant's field of view, and participants wore noise-canceling earplugs to eliminate auditory cues. Physiological recording equipment was placed ~1.5 m from the patient to reduce pump noise without compromising signal quality.

2. Paradigm specificity and generalization

Our IRDT paradigm used a metronome to maintain consistent respiratory timing and encouraged stable tidal volume through paced, even breathing. However, this setup involved voluntary breathing control and biofeedback, which may have heightened respiratory awareness or facilitated compensatory motor responses. These features—while essential for isolating load-related effects—differ from spontaneous breathing and may shape cortical engagement in ways that limit direct generalization to naturalistic conditions.

Our findings are specific to transient inspiratory loading—a brief, mechanically induced perturbation of breathing. While this approach captures both consciously detected and undetected events, the dynamics observed may not fully extend to other respiratory disruptions such as sustained mechanical loads, hypercapnia, hypoxia, or dyspnea driven by emotional or metabolic factors. These conditions may engage distinct time constants and afferent pathways (e.g., chemosensory, limbic), potentially altering network dynamics or dominant cortical and subcortical nodes. The anterior insula–frontal circuit characterized here is particularly suited to detecting and evaluating mechanical mismatch, but extrapolation to broader interoceptive or homeostatic domains should be done with caution (see Section 7).

3. GC–Behavior Correlations

Figure 4B (left) shows that detection accuracy positively correlated with AIC→OFC cGC strength in the 15–40 Hz band (Pearson $r = 0.501$; $n = 5$ participants with AIC and OFC coverage). Bayesian estimation yielded a posterior mode of 0.622 with moderate negative skew

(–0.727), reflecting some uncertainty due to small sample size. Conversely, Figure 4B (right) shows that perceptual threshold negatively correlated with AIC→OFC cGC ($r = -0.357$; posterior mode = –0.424; skew = 0.698). GC–behavior correlations in other pathways were weaker and non-significant (see fig. S15).

4. Load response dynamics and breath-to-breath variability

We compared load-evoked HFA dynamics across regions (Fig. 3A), accounting for breath-to-breath variability and differences in inhalation duration (31). Across participants, mean inhalation duration was 3.07 ± 0.61 s (CI: 2.75–3.40), closely matching the paced metronome (Fig. S9A–B). Individual durations ranged from 1.15–5.10 s and were slightly shorter in preload (2.97 ± 0.58 s) than load trials (3.17 ± 0.66 s; $p = 0.03$) (see Fig. S10A–B). To stabilize phase alignment, we combined airflow (iAF) and mouth pressure (iMP) into a single inspiratory efficacy index (iEff = iAF / iMP), which was then normalized from 0° to 360° (12 bins). Figure S9C shows binned iEff across all trials ($n = 339$). Example electrodes (Fig. S9E, from S9D and 3A) illustrate these dynamics: AIC activity rose in 5 of 12 bins, while premotor cortex activity remained elevated throughout inhalation. At the population level (Fig. S9F), AIC HFA decayed by -12.2% across the phase cycle, compared to weaker decay in OFC (-3%) and minimal change in other frontal regions (Fig. 3B).

Latency analyses revealed that load-evoked HFA emerged earliest in precentral regions, followed closely by the AIC (Fig. S10C), suggesting rapid preparatory engagement in both motor and insular areas. Despite this temporal proximity, Granger causality confirmed that the AIC exerted dominant directional influence on frontal circuits, supporting a model in which insular signals drive salience-based detection and evaluation even as motor regions rapidly initiate compensatory adjustments to breathing. Such dissociations—where onset latency does not dictate causal influence—have been reported in other cortical systems (68-70). For example, in visual cortex, Granger causality analyses revealed that higher-order areas (e.g., V4, TEO) exert feedback control over earlier regions despite their later response latencies (70).

5. Load-magnitude dependent effects and response saturation

Stronger inspiratory loads produced larger physiological and neural responses, but scaling varied by brain region. In an example AIC electrode (Fig. S12A from Fig. 2B), 1 $cmH_2O/L/s$ loads reduced iAF by just 0.75% ($p = 0.54$) yet increased HFA by 9.2% ($p < 0.001$), whereas 15 $cmH_2O/L/s$ loads reduced iAF by 52.6% and increased HFA by 17.5% (both $p < 0.001$). Notably, HFA gains plateaued or declined beyond ~10 $cmH_2O/L/s$, despite continued rises in iMP (–265%, $p < 0.001$).

To quantify these relationships, we fit load–response functions (LRFs) using curve fits similar to approaches for contrast response functions (CRFs) in primary visual cortex (71). Example AIC LRFs confirmed this saturation pattern: HFA rose steeply up to ~10 $cmH_2O/L/s$, then flattened or declined (Fig. S12B). Despite some inter-participant variability, this trend held across the AIC population (Fig. S12C). In contrast, OFC responses scaled more linearly across the full range (Fig. S12D). Saturation indices captured this divergence: AIC and IFGop showed the strongest early saturation, while other frontal, parietal, and cingulate regions exhibited steadier scaling (Fig. S12E). Across all load-responsive sites passing goodness-of-fit (231/239 electrodes), most LRFs plateaued or declined at higher loads (Fig. S12F), indicating nonlinear scaling as a general property of cortical load-responsive regions, despite differences in saturation profiles across areas (AIC > frontal).

While it would have been ideal to separately model load–response slopes for detected versus missed trials (Fig. S7C), the limited number of trials per load level precluded this. As the first iEEG study of its kind—using a design optimized to minimize expectancy and variability—each trial was necessarily lengthy, and most patients fatigued after ~40–50 trials. Addressing finer questions like detection-dependent load tuning will therefore require future paradigms tailored for higher trial throughput.

6. Amygdala coverage

The amygdala was not a focus of this study due to limited electrode coverage and a lack of consistent load-evoked responses in the available sites. However, the amygdala's established role in fear-related respiratory suppression (50), emotional modulation of respiratory drive (35), and apnea (12) warrants acknowledgment. Its omission here does not exclude its involvement in other paradigms (e.g., threat-induced breath-holding, affective dyspnea). Future work with targeted sampling in the amygdala and limbic-affiliated regions may clarify how emotional and affective load dimensions interact with mechanosensory detection.

7. Primary vs. secondary interoceptive cortices and relevance to hierarchical models

In line with recent neuroimaging studies (15, 47, 48), cortical load processing was observed not only in the anterior insula (AIC) and orbitofrontal cortex (OFC), but also in frontomotor, posterior insula (PIC), cingulate, and parietal regions (Figs. 1F, 2E). Within somatosensory cortex (postcentral gyrus), 36% of electrodes responded to loads with modest HFA increases (5.3% vs. AIC: 9.2%; Fig. 1G). Perceptual gain effects were present but weaker in somatosensory and adjacent parietal regions (27.3% vs. AIC: 54.5%, OFC: 41.6%), and activity in these regions instead tracked perceived load intensity (Fig. 7B), consistent with a role in sensory discrimination rather than detection per se.

Although load and perceptual gains in PIC were modest, this region uniquely exhibited Granger-causal influence on AIC during loaded trials (Figs. 3E and S8C), suggesting early sensory processing or gating upstream of anterior insula engagement. In addition, load responses in missed trials showed a distinct anatomical distribution, with relatively stronger gains outside the AIC—particularly in the anterior cingulate cortex (Fig. S7B)—and were less sensitive to load magnitude than in detected trials (Fig. S7C).

Together, these findings are consistent with a functional gradient from primary sensory encoding to detection- and appraisal-related processing. However, electrode sampling across primary and secondary interoceptive cortices was sparse or uneven, limiting finer-grained hierarchical inferences. Moreover, confidence and metacognitive ratings were not collected, further constraining interpretation within formal hierarchical interoceptive models.

8. Control analyses for breath order, expectancy, timing, and muscle artifacts

To complement the main Results, we provide additional methodological detail for the control analyses used to rule out nonspecific effects of breath order, anticipatory buildup, motor timing, and EMG contamination.

Breath-position and expectancy controls. Because inspiratory loads were applied on later breaths within a trial, we reanalyzed the data by comparing loaded inhalations to no-load trials matched for breath position across trials, rather than only to the immediately preceding preload. Example electrodes showed comparable baseline HFA for preload and matched-position no-load breaths (Fig. S13A). Across electrodes and regions, this matched-position analysis yielded nearly identical spatial coverage, and HFA gains relative to the preload contrast (Fig. S13B–C), confirming that load effects are not driven by breath number or expectancy alone.

Pre-inspiratory excitability across breaths. To test whether baseline cortical excitability drifted across the paced sequence, pre-inspiratory HFA was quantified in a −1 to 0 s window relative to inhalation onset, normalized to breath 1, and evaluated across breaths. No significant breath-order effects were observed within any region or when averaging across regions (Fig. S13D), indicating stable baseline excitability.

Motor timing control. Cue-to-inhalation latency was computed as the interval between visual cue onset and inspiratory flow onset. Latencies did not differ across breaths at either the individual or group level (Fig. S13E), ruling out systematic changes in motor readiness or task engagement.

Control for muscle artifacts. To minimize potential contamination of broadband HFA by orofacial or neck muscle activity, all key analyses were repeated using a narrower gamma band (35–75 Hz). Load-related and perception-related effects were preserved with similar magnitude and spatial distribution (Fig. S14), supporting a cortical rather than muscular origin.

Together, these control analyses demonstrate that the reported HFA, coherence, and Granger causality effects reflect genuine cortical processing of inspiratory load, rather than nonspecific effects of breath order, expectancy, timing, or EMG contamination.

Fig. S1

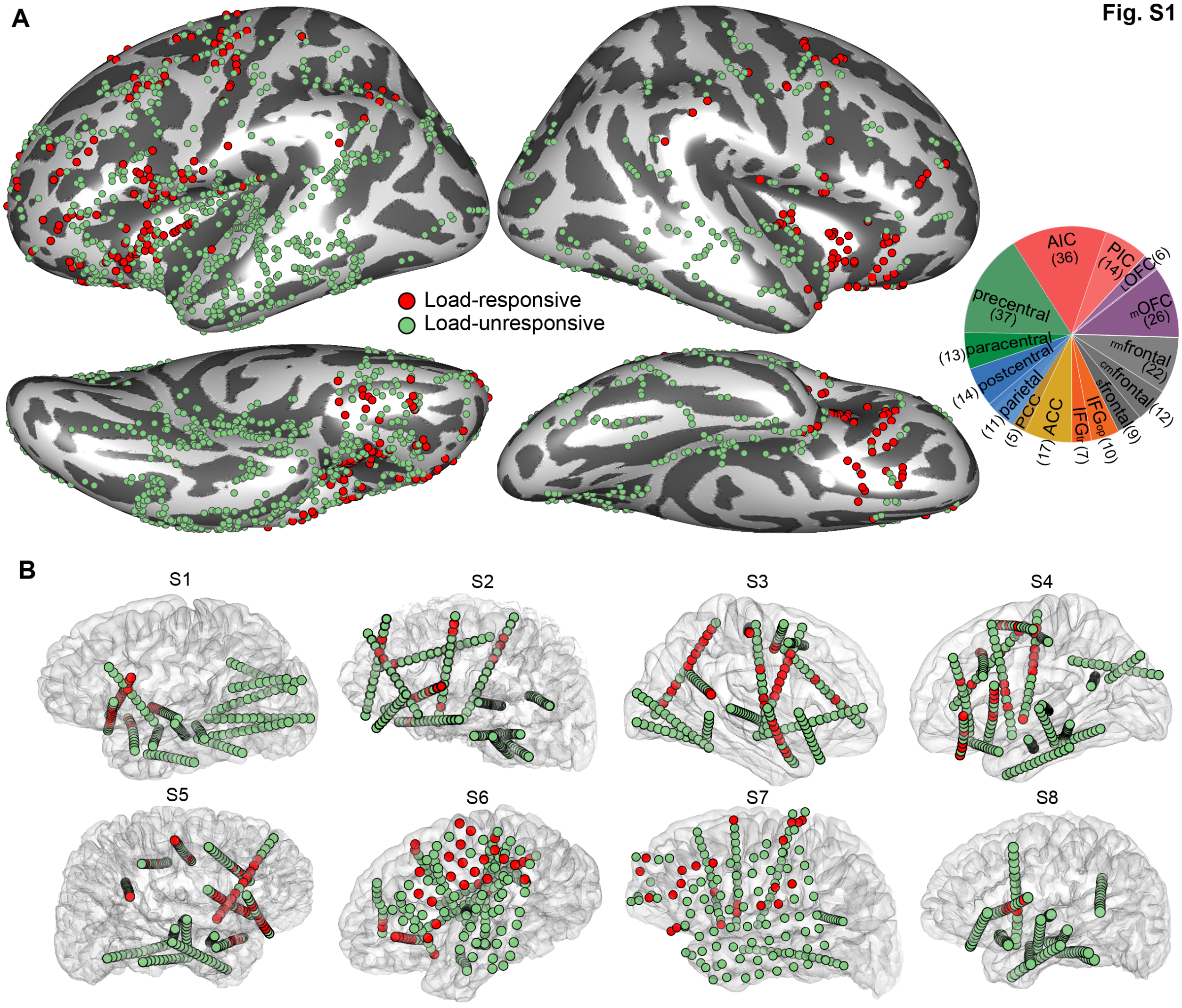

Figure S1. Electrode coverage and load effects.

(A) Load-responsive (red) and unresponsive (green) electrodes from all 8 participants overlaid on the FreeSurfer average inflated surfaces. Of 1328 electrodes, 239 (18%) showed significant load responses (greater HFA in loaded vs. preloaded inhalations, 0–3 s post-inhalation onset). Pie chart shows the regional distribution of these 239 load-responsive electrodes; numbers in brackets indicate the total number of electrodes sampled in each brain region.

(B) Same as A, but shown separately for each participant. Translucent pial surface plots display both epidural and deeper electrodes on the individual brain. Most representative hemisphere is shown for each participant. Participants 3 and 4 had some electrodes in the contralateral hemisphere (not shown). Participants 6 and 7 were implanted with both depths and grids. Electrode contacts are enlarged for visibility.

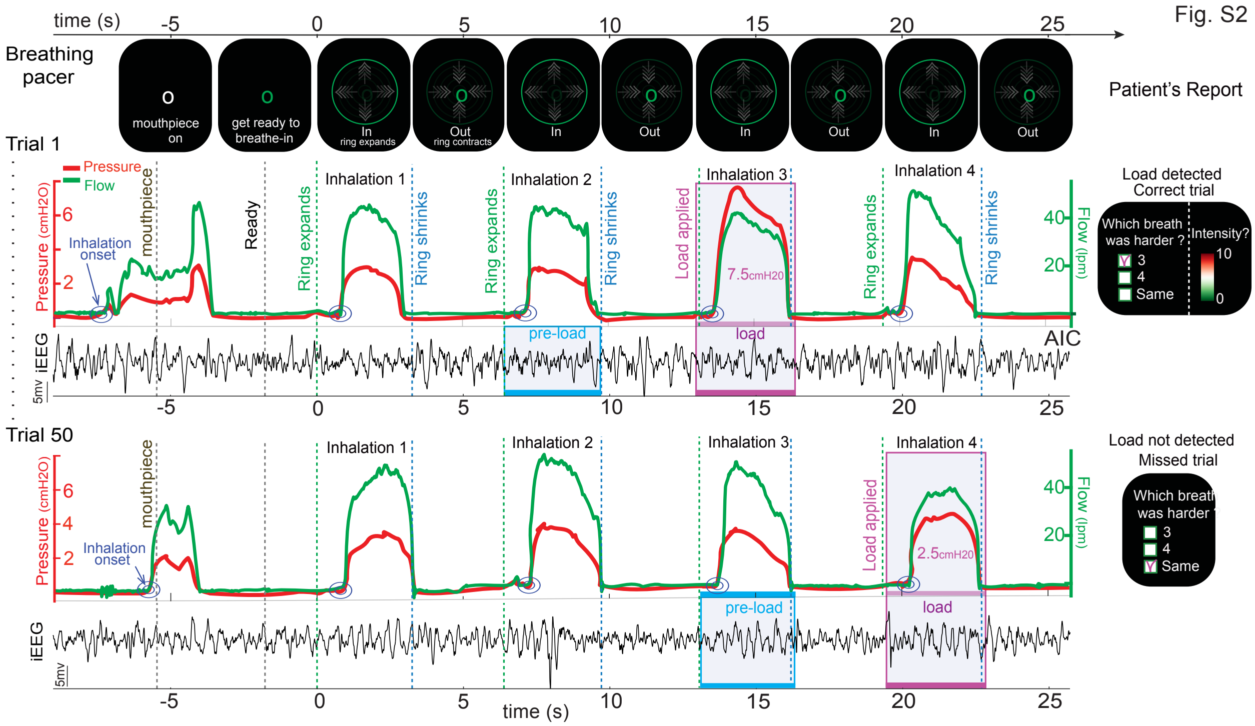

Figure S2. Example IRDT trials with respiratory and neural recordings.

Two example trials from the Inspiratory Resistance Detection Task (IRDT), where patients took four paced breaths (3 s inhale, 3 s exhale) guided by an expanding/contracting ring (green/blue dashed lines) and reported if a load was applied and its intensity. Loads were unpredictably delivered on one inhalation per trial. Traces show inspiratory airflow (iAF), mouth pressure (iMP), and HFA from an anterior insular cortex (AIC) electrode. Top: detected 7.5 $cmH_2O/L/s$ load. Bottom: undetected 2.5 $cmH_2O/L/s$ load. Blue dots mark inhalation onsets.

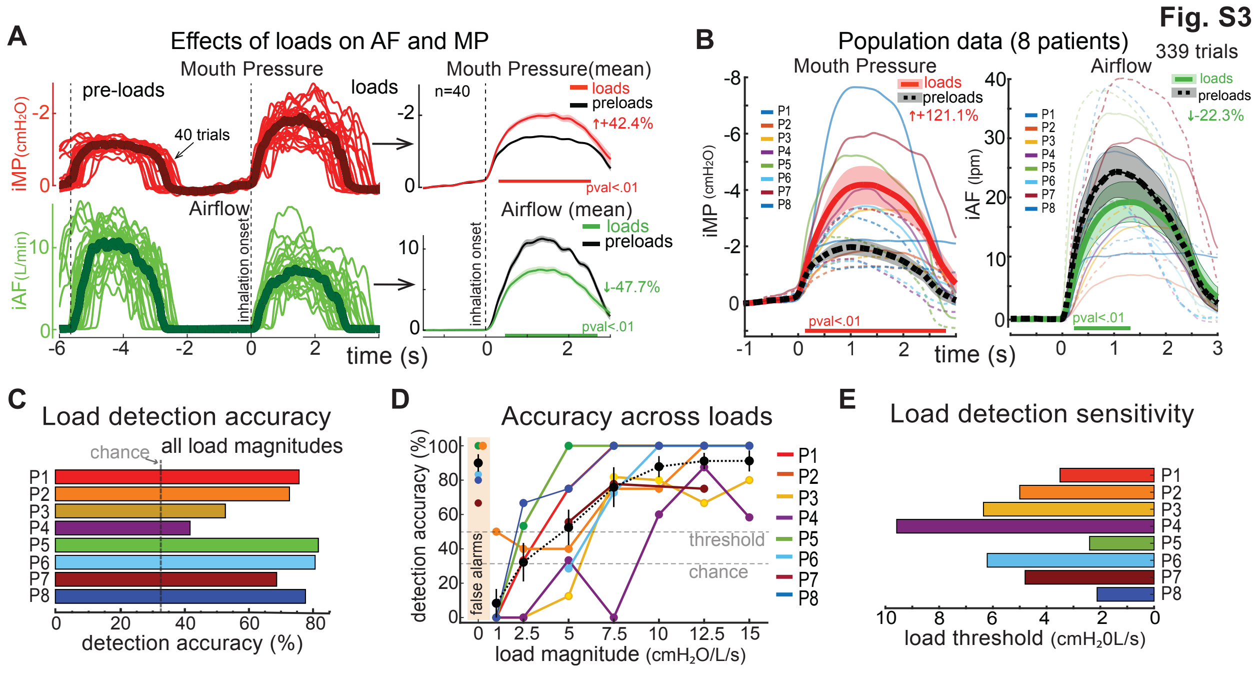

Figure S3. Load-dependent changes in inspiratory effort and detection accuracy.

(A) Inspiratory mouth pressure (iMP, red) and airflow (iAF, green) from an example participant across 40 trials (7 load magnitudes). iMP rose and iAF fell during loaded vs. preloaded inhalations (0–3 s post-onset). Thin lines show single trials; thick lines show means. Right: overlaid mean traces ± SEM.

(B) Group iMP (left) and iAF (right) across all participants (n = 8; 339 trials). Thin colored lines indicate participant means; thick dashed lines show grand averages.

(C) Overall detection accuracy. Bars indicate percent correctly detected trials across all loads; dashed line marks chance (33%).

(D) Detection accuracy by load magnitude for each participant.

(E) Detection thresholds, defined as the lowest load detected >50% of the time.

Fig. S4

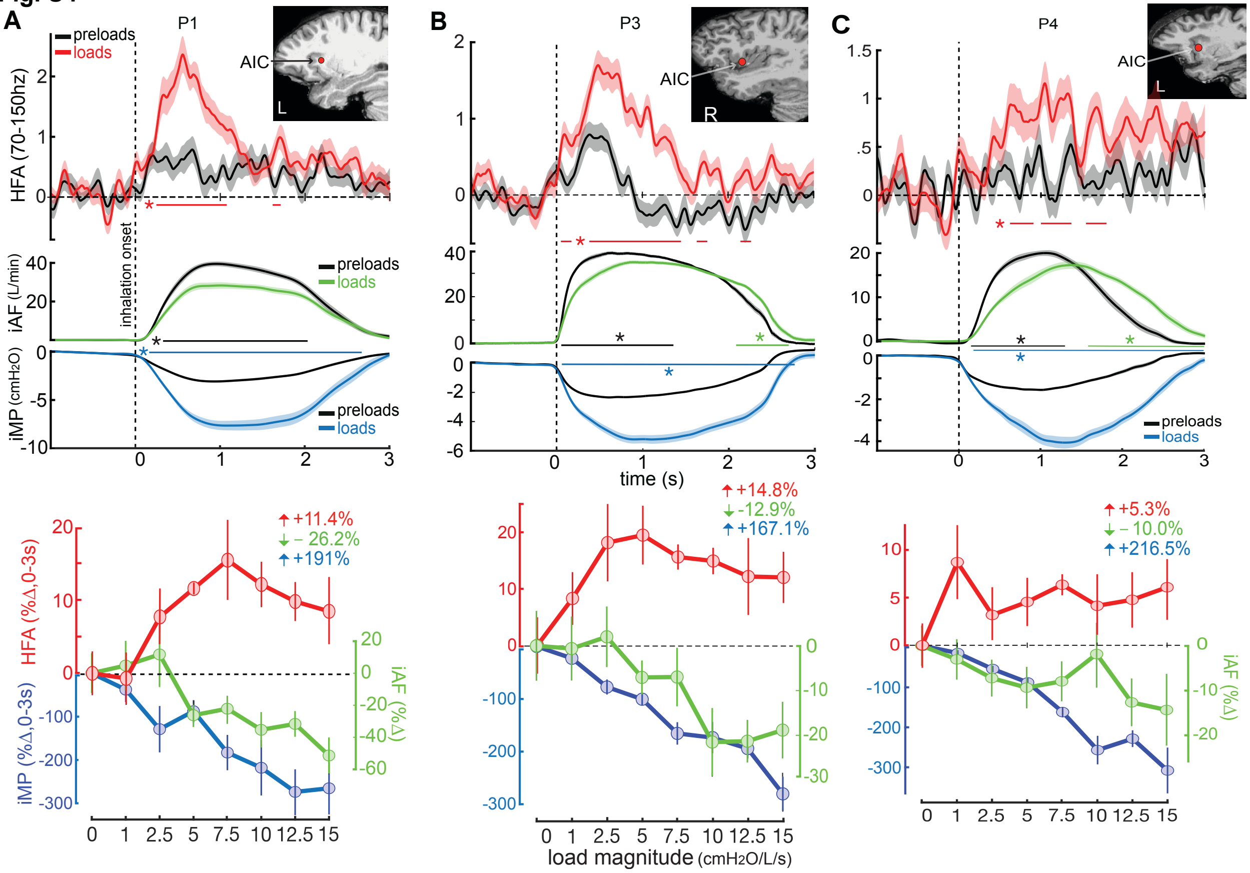

Figure S4. Load-responsive electrodes in the anterior insular cortex (AIC).
Three representative AIC electrodes from different participants showing load-evoked neural and respiratory responses.
(A) Left AIC in participant S1 (48 trials).
(B) Right AIC in participant S3 (42 trials).
(C) Left AIC in participant S4 (38 trials).
Each panel shows (left) time courses of HFA (70–150 Hz; top), inspiratory airflow (iAF; green), and mouth pressure (iMP; blue) averaged across loaded (red) vs. preloaded (black) inhalations, and (right) mean responses as a function of load magnitude (0–3 s post-inhalation). HFA rose with load alongside increased inspiratory effort (iMP, iAF). Asterisks indicate significant differences from preloads ($p < 0.05$, ranksum). Shaded areas denote SEM. Vertical dashed lines mark inhalation onset.

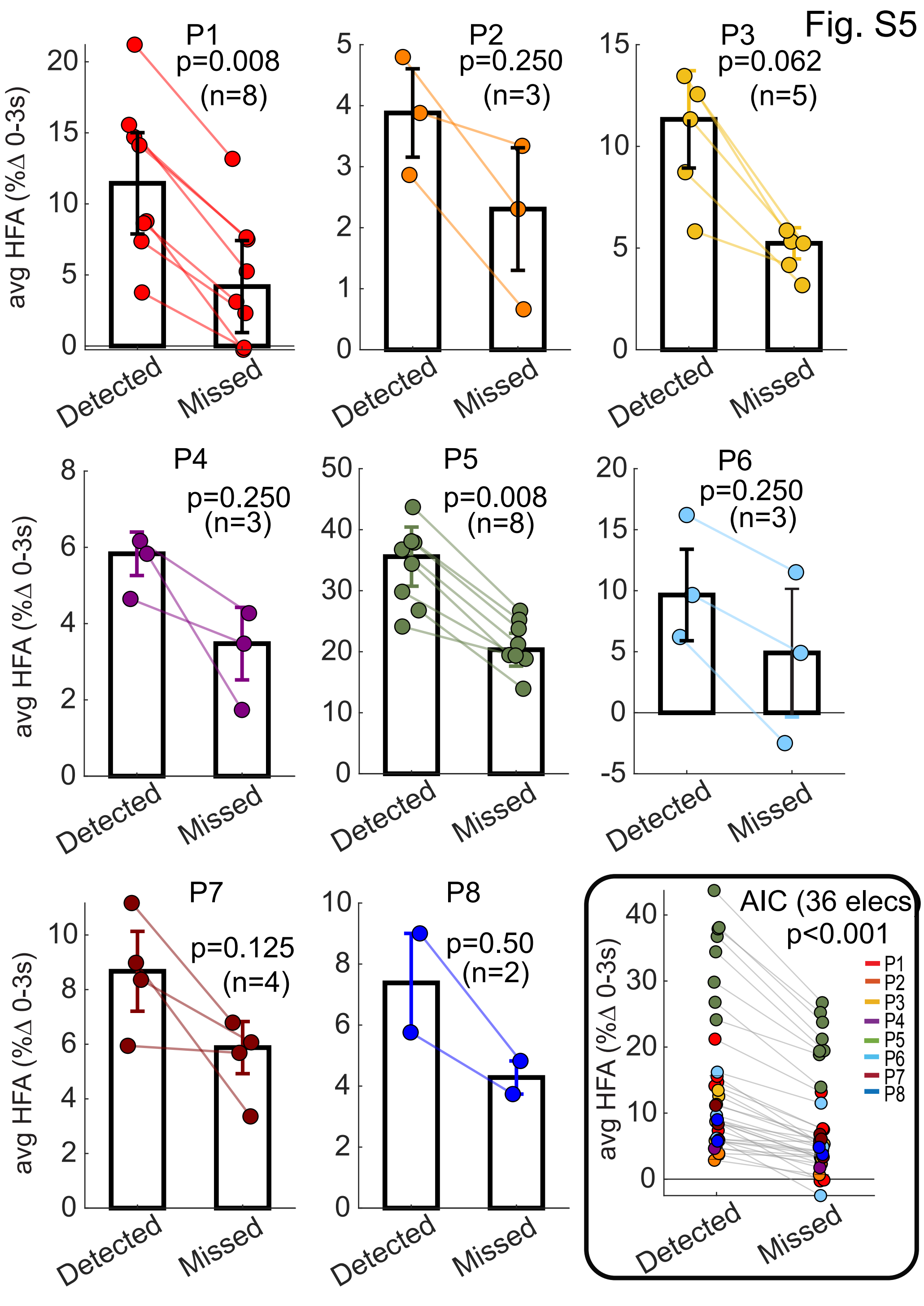

Figure S5. Individual AIC responses to detected versus missed loads.
Mean HFA gains (0–3 s post-inhalation) in the anterior insular cortex (AIC) for detected vs. missed trials, plotted separately for each participant (n = 8). Trials were matched by load magnitude. Bars show mean ± SEM. (Inset) Group-averaged HFA gains across all AIC electrodes (n = 36).

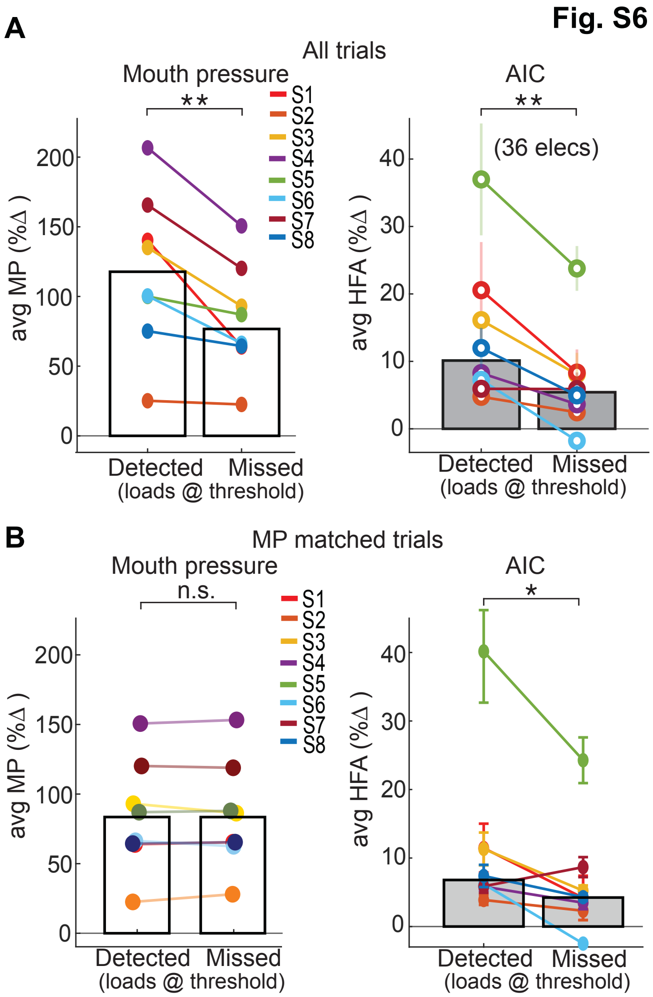

Figure S6. Mouth pressure differences in detected vs. missed trials.
(A) All trials: Inspiratory mouth pressure (iMP, left) was significantly higher for detected versus missed trials of the same magnitude ($p = 0.008$; Wilcoxon signed-rank), while AIC HFA gains (right, 36 electrodes) were also significantly greater in detected trials.
(B) After matching trials to control for iMP (no significant pressure difference), HFA gains in the AIC remained higher for detected loads of the same magnitude ($p = 0.039$), indicating that increased respiratory effort did not fully explain neural differences. Each line shows data from one participant (colors), bars indicate group mean ± SEM.

Fig. S7

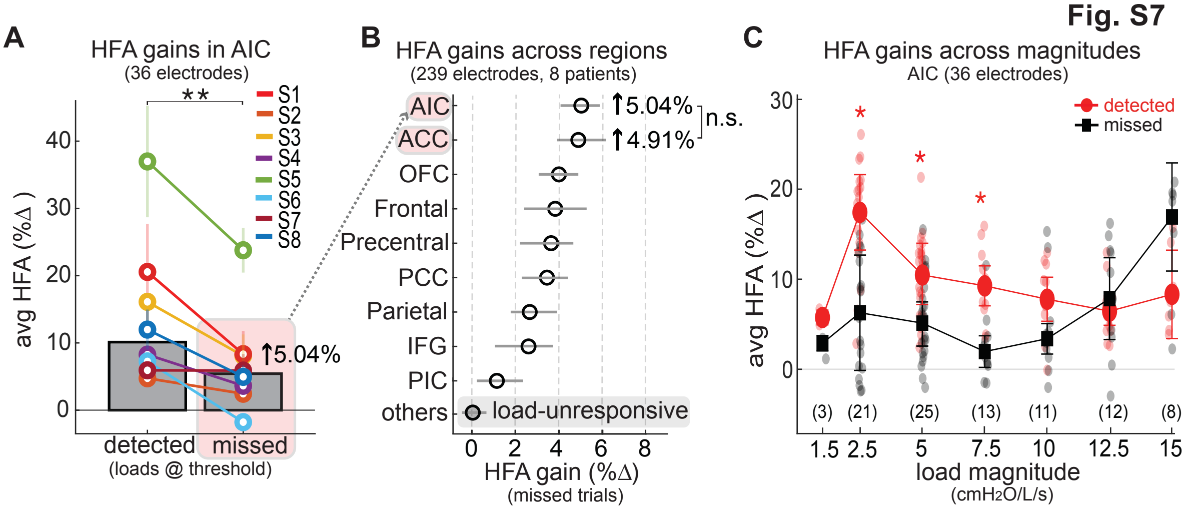

Figure S7. Neural responses during missed load trials.
Even without detection, modest AIC HFA gains indicate subthreshold processing.
(A) AIC electrodes showed small but significant HFA increases (~5%) during missed trials.
(B) These effects were most prominent in the AIC and ACC.
(C) HFA gains in detected trials peaked at lower loads (where detection is critical), while missed-trial gains increased more for higher loads. Numbers denote electrode counts per magnitude.
Abbreviations: HFA = high-frequency activity; OFC = orbitofrontal cortex; PCP = postcentral/parietal cortex; SMA = supplementary motor area; PMC = premotor cortex; ACC = anterior cingulate cortex; AIC = anterior insular cortex.

Fig. S8

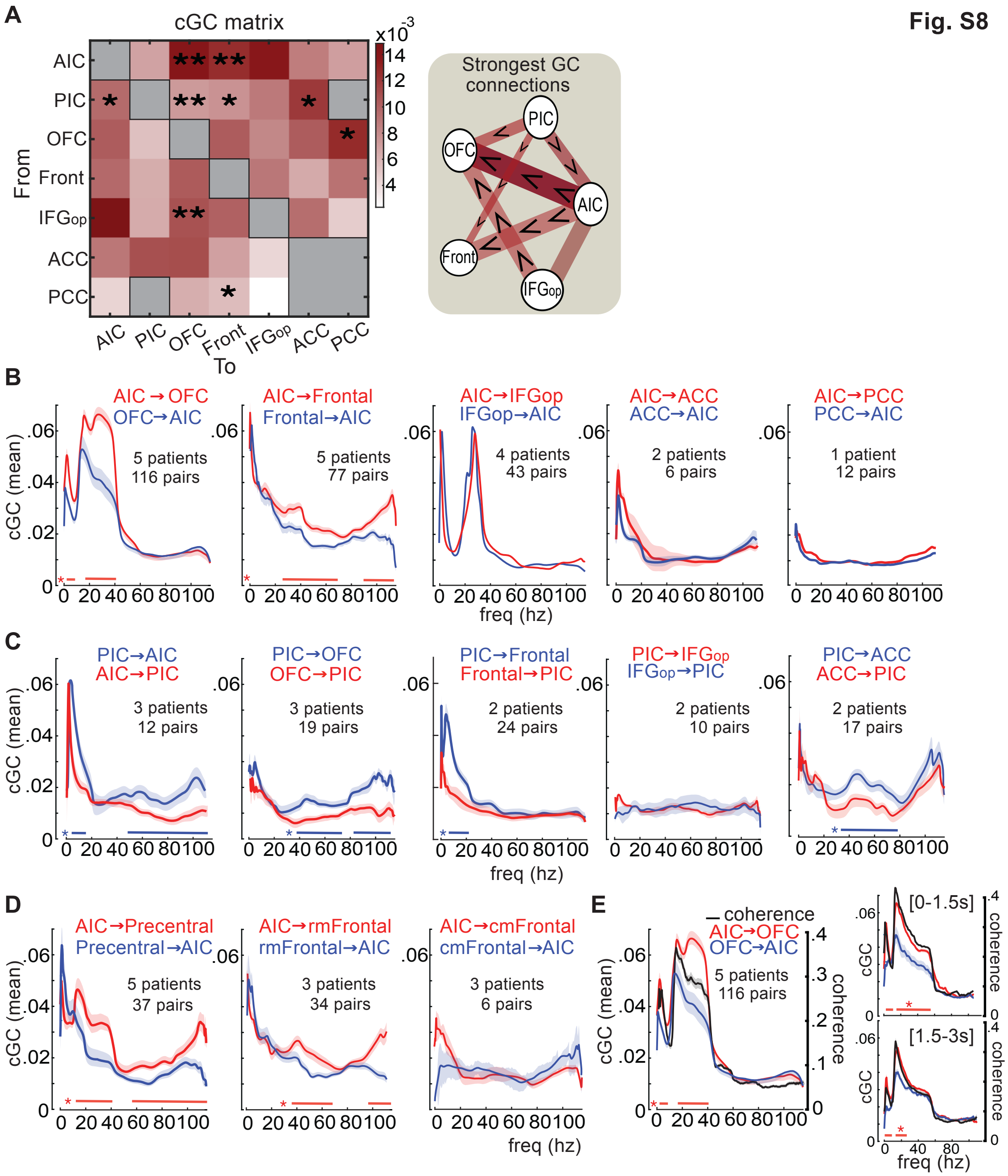

Figure S8. Connectivity profiles supporting load detection.

(A) Conditional Granger causality (cGC) matrix across load-responsive brain regions during loaded trials (0–3 s post-inhalation). Darker red indicates a larger absolute GC estimate; asterisks mark where the GC in one direction is significantly higher than the reverse, indicating directional asymmetry. Right: schematic summarizes dominant connections (arrow direction, line width/color denote strength).

(B) Spectral-resolved cGC from AIC to target regions, showing a distinct peak at 15–40 Hz for AIC→OFC and a broader elevation spanning 25–115 Hz for AIC→frontal cortex. Asterisks along the x-axis indicate frequency bins where GC directionality (AIC→target vs. reverse) was significant. GC was computed for each electrode pair (e.g., AIC#1 and OFC#1 in P1, AIC#1 and OFC#2 in P1, and so on across all participants) and then pooled to generate these curves.

(C) Spectral-resolved cGC from PIC, showing broader but weaker influence than AIC.

(D) AIC→frontal cGC separated by subregion, strongest for precentral cortex.

(E) AIC→OFC coherence peaked at 18 Hz, matching GC frequencies, and was reduced (but not absent) in later inhalation phases (1.5–3 s vs. 0–1.5 s).

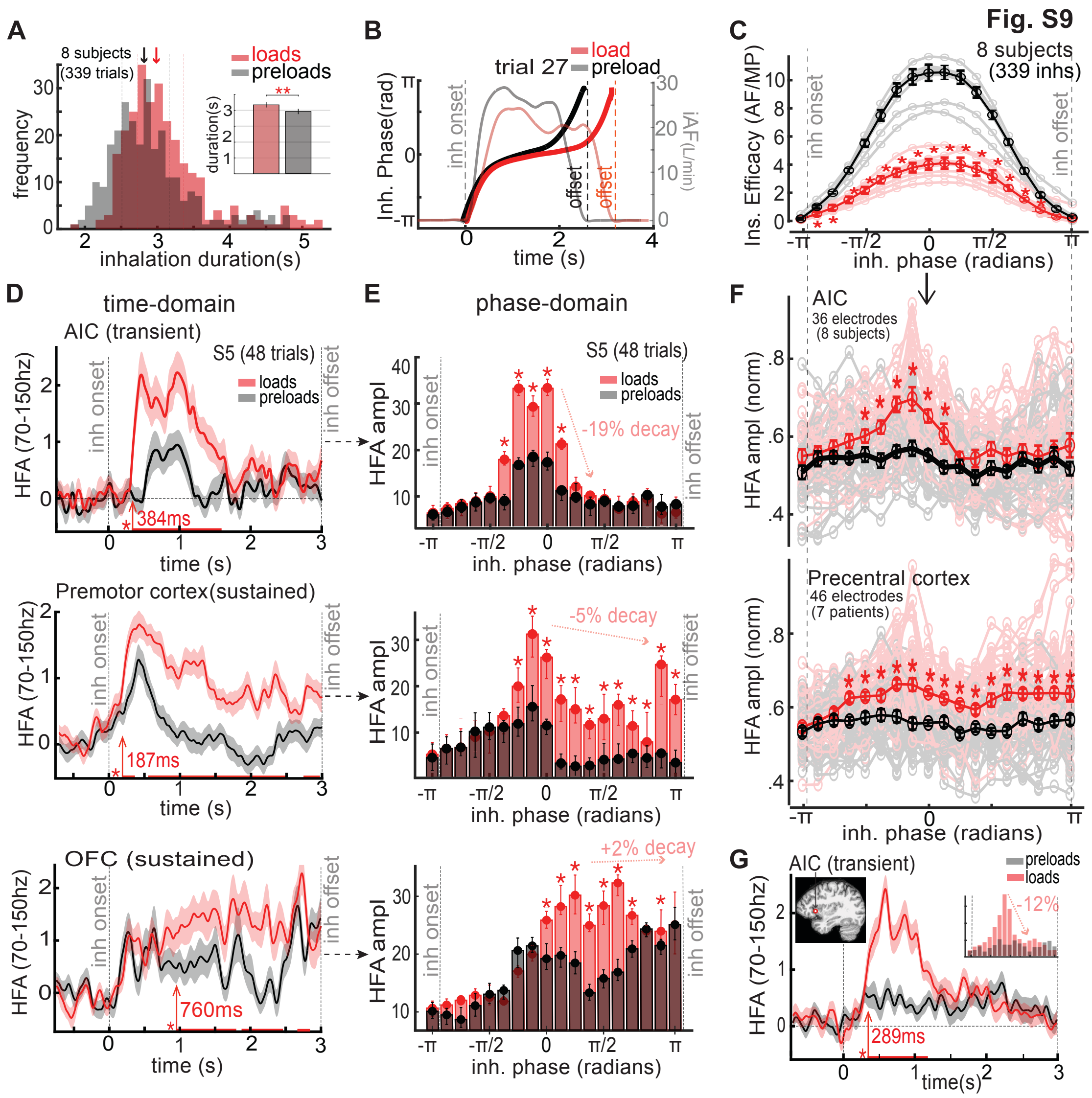

Figure S9. Transient vs. sustained load responses and phase-aligned HFA dynamics across cortical regions.
(A) Inhalation durations for all preloaded and loaded trials (n = 339; 8 participants). Preloaded inhalations were slightly shorter ($p < 0.01$); inset shows group means ± 95% CI.
(B) Example trial showing conversion of airflow to inhalation phase. Each inhalation was normalized from 0–360° (–π to π radians) to account for duration variability.
(C) Phase-aligned inspiratory effort index (iAF/iMP) across all participants and trials. Each line shows the averaged IE for one participant. Preloaded trials showed reduced effort, peaking near mid-inhalation. Asterisks indicate significant bin differences.
(D) Time-domain HFA (70–150 Hz) responses from AIC, premotor, and OFC electrodes in one participant (S5; 48 trials, 7 load magnitudes). AIC responses were transient, whereas frontal regions showed sustained activity. Red arrows mark load-related response latencies.
(E) Phase-domain HFA from the same electrodes. AIC showed brief early increases, while premotor and OFC responses extended across the inhalation. Each bin shows the median and interquartile range across trials. Asterisks indicate significant bins (signed-rank, $p < 0.05$).
(F) Population phase-binned HFA for all AIC (n = 36) and precentral (n = 46) electrodes. Asterisks mark significant bins ($p < 0.05$).
(G) Time- and phase-domain HFA for the AIC electrode shown in Fig. 2B.

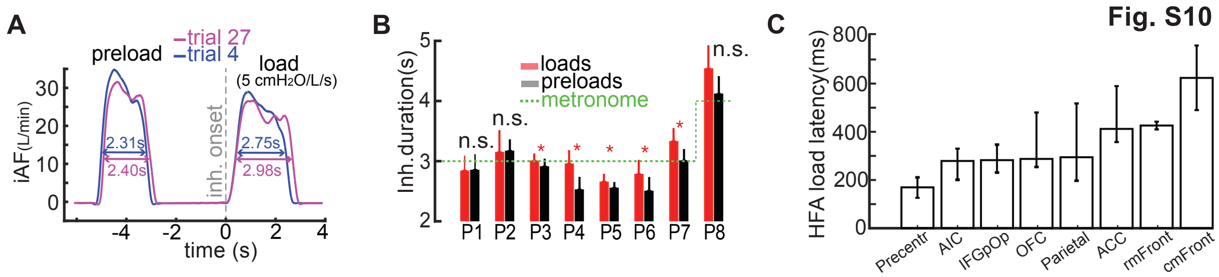

Figure S10. Breath-to-breath variability and latency of neural responses.
(A) Example trials showing breath-to-breath variability in inhalation duration across preloaded and loaded conditions.
(B) Inhalation durations by participant and condition. Bars indicate mean ± SE across trials. Dashed green line marks the pacer target (3 s inhalations for all participants except Participant 8, who used 4 s to better match her natural breathing pace).
(C) Latency of load-related HFA increases across cortical regions. Bars show median onset latency for significant HFA elevations (load vs. preload). Motor areas (precentral, including motor, premotor, and SMA) responded earlier than AIC and OFC, suggesting rapid motor engagement during inspiratory load processing. See Fig. S9D for single-electrode examples.

Fig. S11

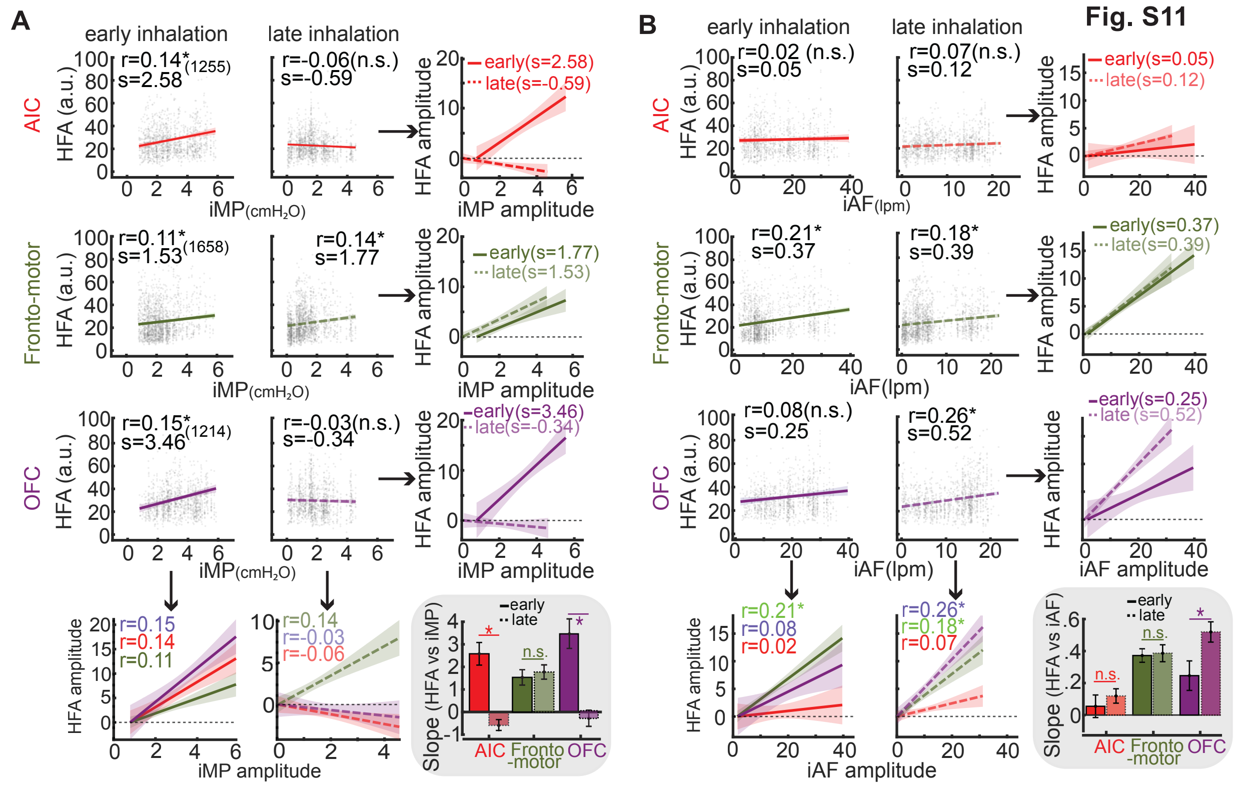

Fig. S11. Distinct cortical encoding of inspiratory pressure and airflow across inhalation phases.

(A) Trial-level correlations between high-frequency activity (HFA) and inspiratory mouth pressure (iMP) amplitude were computed during early and late inhalation in the anterior insula (AIC), fronto-motor cortex, and orbitofrontal cortex (OFC). The AIC selectively encoded pressure in the early phase. Fronto-motor regions showed significant correlations in both phases. OFC exhibited prominent early-phase sensitivity to pressure. Each subplot shows Pearson correlation (r), slope (s), and individual trial data. In plots with overlaid regression lines (arrows), HFA was aligned to zero at the minimum iMP to facilitate slope comparison. Summary plots (grey box) compare slope differences across regions and phases. Asterisks indicate significant Pearson correlations or significant slope differences based on signed-rank tests after FDR correction.

(B) Same analysis as in (A), using inspiratory airflow (iAF). Fronto-motor regions again showed robust, phase-invariant modulation. The AIC exhibited weak airflow encoding. OFC responses were minimal in the early phase but increased in the late phase, with higher correlation and slope values. Summary plots (grey box) illustrate signal- and phase-specific encoding across regions.

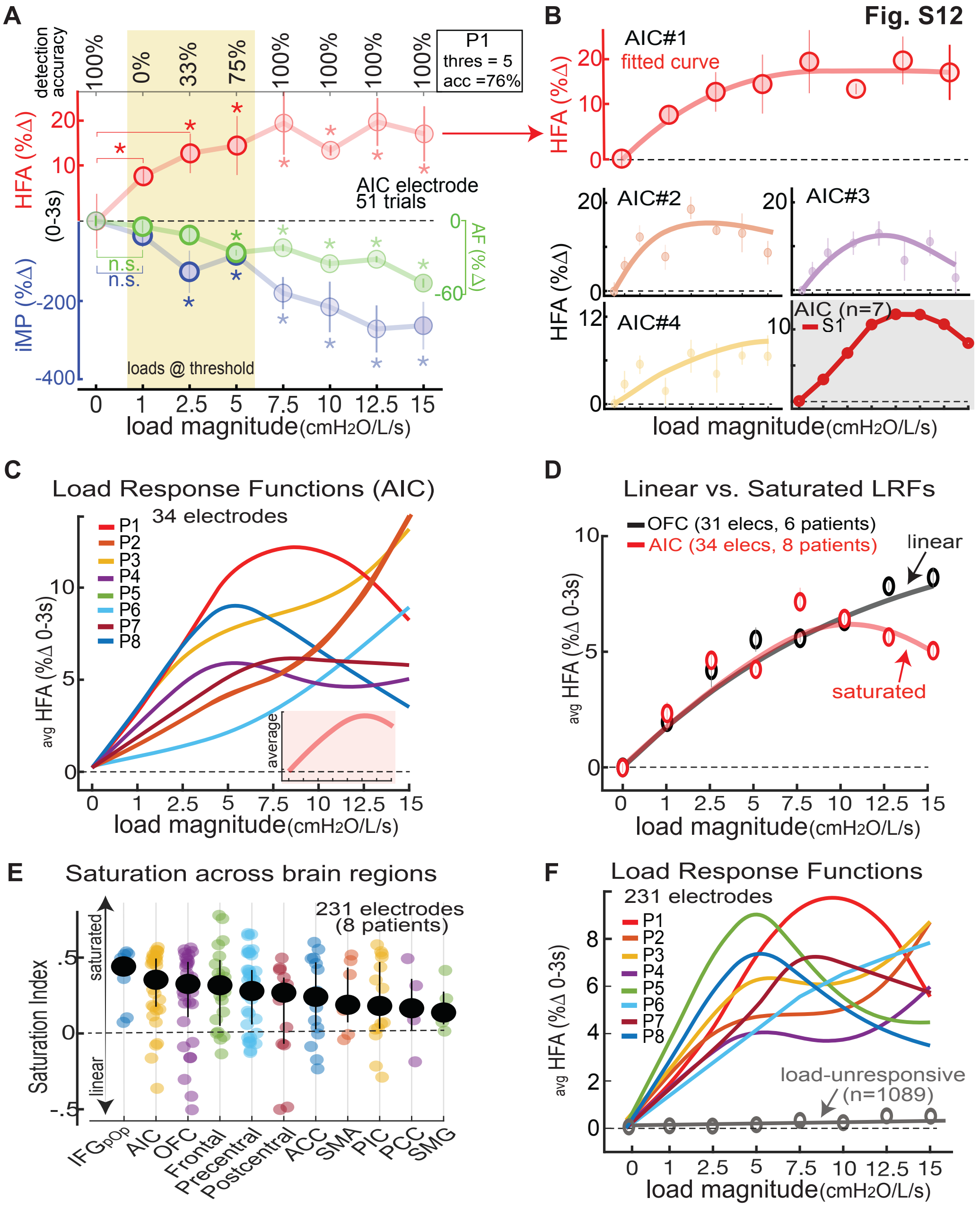

Fig. S12. Load-magnitude tuning and saturation across cortical regions.

(A) Same AIC electrode as Fig. 2B, showing HFA (red), inspiratory pressure (iMP, blue), and airflow (iAF, green) across increasing load magnitudes. HFA rose up to ~10 cmH₂O/L/s, then plateaued or declined. Detection accuracies are marked above.

(B) Top: Curve fit to mean HFA from this electrode. Bottom: Load-magnitude response functions (LRFs) from three AIC electrodes in the same participant, all showing saturation. Right: Averaged LRF across all seven AIC electrodes from this participant (one electrode failed goodness-of-fit and was excluded).

(C) LRFs from AIC electrodes across eight participants (34/36 electrodes passed the goodness-of-fit test).

(D) Participant-averaged LRFs in AIC and OFC. AIC responses saturated early, whereas OFC responses increased more linearly.

(E) Saturation index by region. Each dot represents one electrode; black circles show regional medians ± IQR. AIC and IFGop showed the strongest saturation.

(F) Load-magnitude response profiles (LMRs) from all significantly load-responsive electrodes ($n = 231$; eight electrodes excluded for poor fit). Most showed plateauing or declining HFA at higher loads. Dashed gray line shows the average response across load-unresponsive electrodes ($n = 1089$).

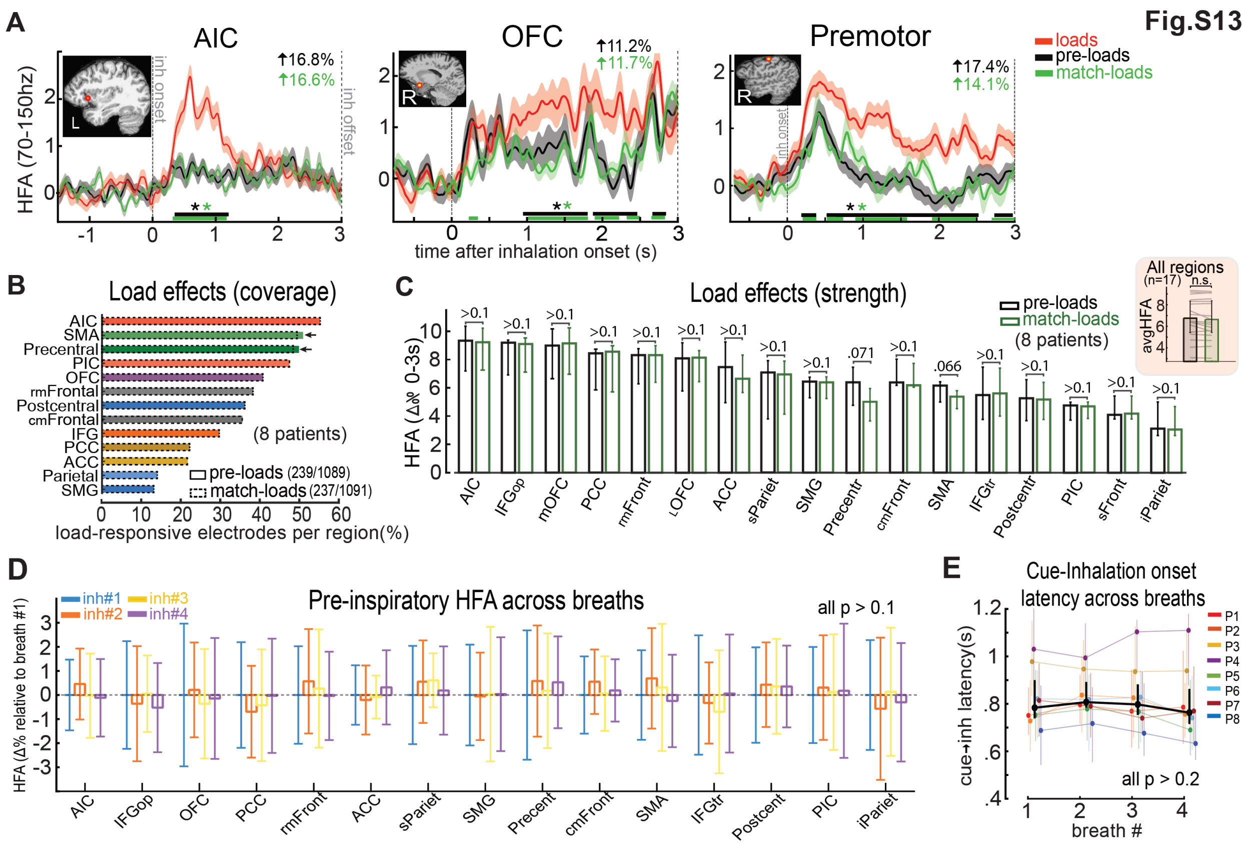

Fig. S13. Control analyses rule out nonspecific effects of breath order, expectancy, and timing.

(A) Example electrodes showing similar inspiratory-evoked HFA for preload and matched-position no-load inhalations, resulting in comparable baseline activity relative to loaded inhalations. Percent values indicate mean load-evoked HFA increases relative to preload or matched-position no-load baselines (0–3 s post–inhalation onset).

(B) Coverage analysis comparing load-responsive electrode distributions obtained using preload versus matched breath-position controls, showing similar spatial patterns across cortical regions.

(C) Comparison of load-evoked HFA strength using preloaded breaths versus matched breath-position no-load controls. No significant difference in HFA gain was observed when averaging across regions (Wilcoxon signed-rank test; $p > 0.1$; inset), nor within any individual region.

(D) Pre-inspiratory cortical excitability across breaths. HFA measured in the −1 to 0 s window before inhalation onset, normalized to breath 1, across four successive breaths and regions. No significant breath-order effects were observed within any region or when averaging across regions (all Friedman tests $p > 0.1$).

(E) Cue-to-inhalation latency across breaths for individual participants (P1–P8) and group average. Latencies did not differ across breaths, indicating stable behavioral timing throughout the trial (all $p > 0.2$).

Together, these control analyses indicate that load-related HFA effects are not driven by breath order, anticipatory buildup, or timing differences, supporting their interpretation as genuine cortical responses to inspiratory load.

Fig. S14

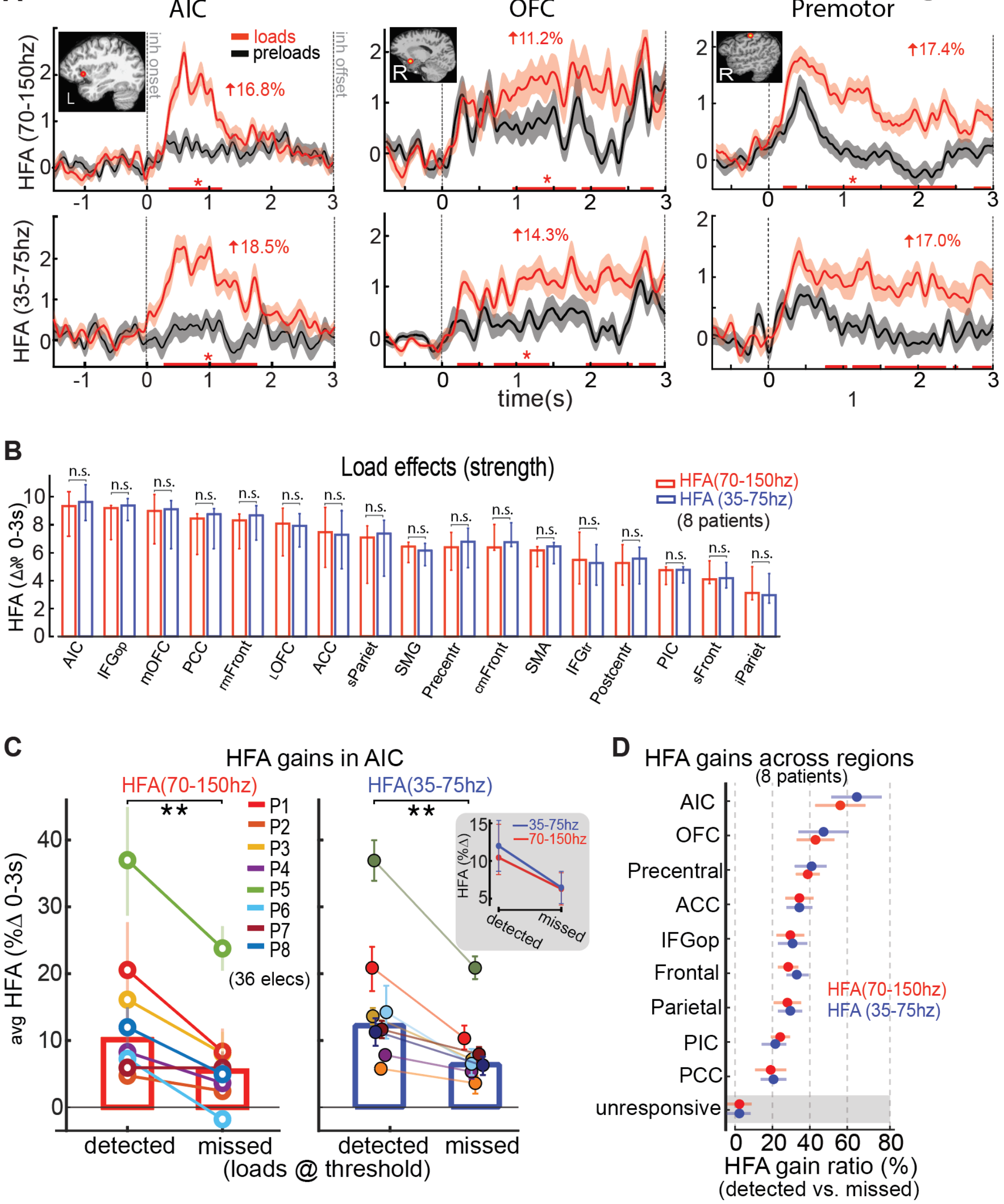

Fig. S14. Control for potential EMG contamination using narrow-band gamma.

(A) Example electrodes in anterior insula (AIC), orbitofrontal cortex (OFC), and premotor cortex showing load-evoked activity plotted using both broadband HFA (70–150 Hz; top) and narrow-band gamma (35–75 Hz; bottom). Load-related responses are preserved in the narrow band, with comparable temporal profiles and relative increases (percentages indicate mean HFA increase during loaded vs. preload inhalations, 0–3 s post–inhalation onset).

(B) Regional load effects on HFA strength computed using broadband (70–150 Hz) and narrow-band (35–75 Hz) gamma. Across all regions, load-related increases did not differ significantly between frequency bands (all n.s.), indicating that observed effects are not driven by high-frequency muscle activity.

(C) Perception effects in AIC. HFA gains for detected versus missed trials computed using broadband and narrow-band gamma show a preserved detection-related enhancement in both bands (36 electrodes, 8 participants). Inset shows comparable detected–missed separation across frequency ranges.

(D) HFA gain ratios (detected vs. missed) across cortical regions using broadband and narrow-band gamma. The spatial pattern and magnitude of perceptual effects are similar across frequency bands, including null effects in unresponsive electrodes (all $p > 0.1$).

Together, these analyses demonstrate that the main load-related and perception-related effects persist when restricting analyses to a narrower gamma band, supporting a cortical origin rather than contamination by orofacial or neck muscle activity.

Fig.S15

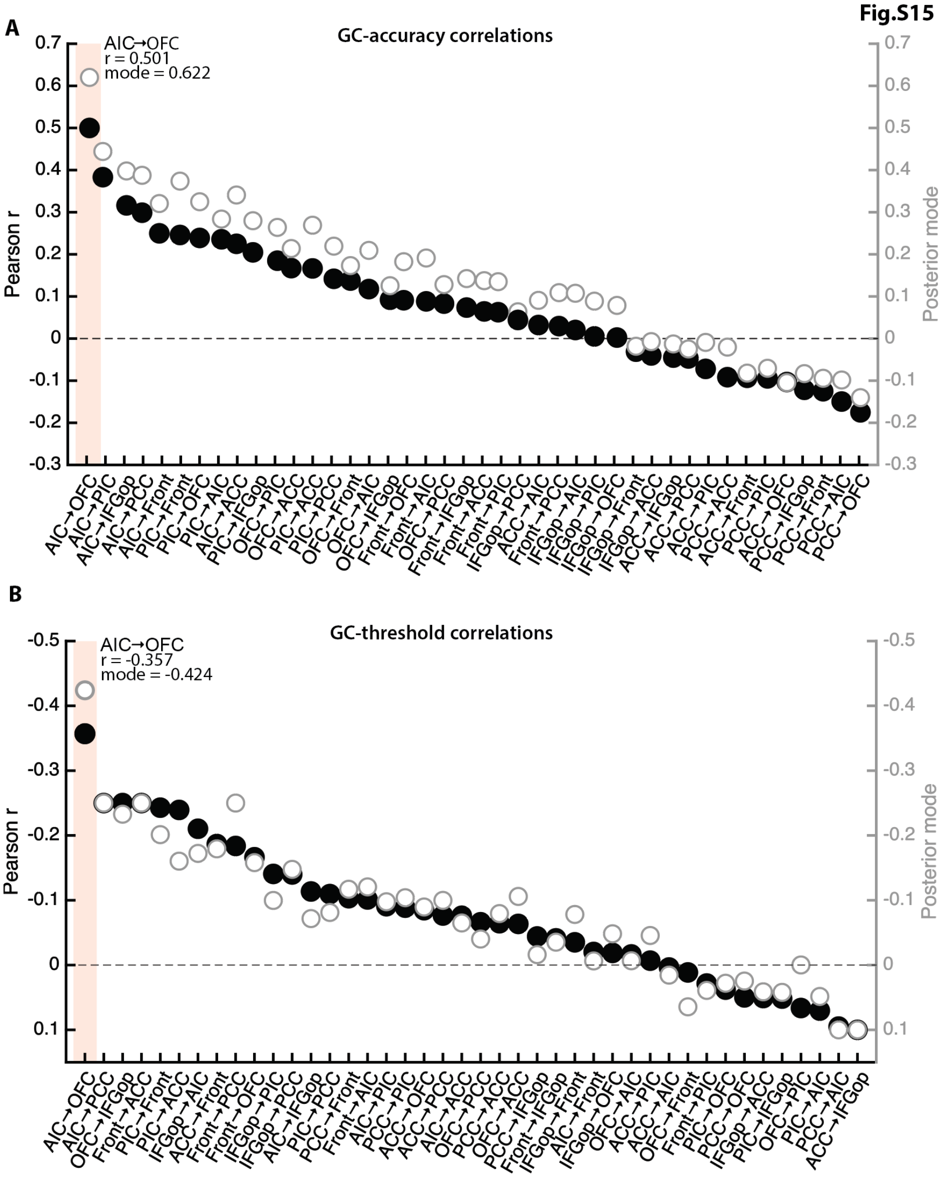

Fig. S15. GC–behavior correlations across directed pathways in the 15–40 Hz band.
(A) Pearson correlations (filled circles) and Bayesian posterior modes (open circles) between detection accuracy and directed cortico-cortical Granger causality (cGC) strength across all tested region pairs. The strongest positive association occurred for the AIC→OFC pathway (Pearson r = 0.501; posterior mode = 0.622; n = 5 participants with AIC and OFC coverage). Thus, participants with stronger AIC→OFC interactions in the 15–40 Hz range tended to detect inspiratory loads more accurately.
(B) Corresponding correlations between perceptual threshold and directed cGC strength. The strongest negative association again occurred for the AIC→OFC pathway (r = –0.357; posterior mode = –0.424), indicating that stronger AIC→OFC coupling was associated with lower perceptual thresholds (i.e., greater sensitivity to respiratory perturbations). Correlations for all other directed pathways were weaker (approximately –0.25 to 0.1), indicating that GC–behavior relationships were selective for the AIC→OFC interaction rather than reflecting a general property of directed connectivity across the network.

ptID	Age	Sex	Brain Anomaly	SOZ	Elecs	Etiology	SOZ - spread
P1	21	M	TO heterotopia	L sup P/O	234 (L/R)	Unknown (sz since 14y old)	Spread to L hippocam pus
P2	59	F	none	L mid-sup T	280 (R)	Unknown (sz since 50y old)	focal
P3	43	F	Multifocal encephalopathy	R T operculum	153	stroke	R O/P
P4	33	M	none	L amygdala	249	Perinatal stroke	hippocam pus
P5	18	M	none	R mesial T lobe	215	unknown	T pole, OFC
P6	24	F	cortical dysplasia	multifocal	167	heterotopia in L F/P	Perisylvian
P7	25	M	none	L lateral T	223	unknown	L F
P8	26	F	none	L mid/inf T	237	unknown	L Mesial T
P9*	50	F	R temporal cavernoma	R Insula	218	unknown	R T operculum
P10*	31	M	L post T cavernoma	L mes T	161	unknown	OFC
P11*	55	F	none	L hippocampal	216	unknown	L T tip

Table S1: Patient seizure information

T = temporal; O = occipital; P = Parietal. F = Frontal. Electrodes = total electrodes implanted across both hemispheres; L = left hemisphere; R = right hemisphere; s = superior (e.g., sP = superior parietal); SOZ = seizure onset zone; Sz = seizures. Asterisk (*) indicates patients excluded from main analyses due to task non-compliance or excessive epileptogenic discharges within targeted (interoceptive) brain regions. Participant 9: Excluded due to right mid-insula SOZ with frequent interictal spikes (>5/min) and ictal spread to the temporal operculum and adjacent inferior frontal regions, including OFC; also failed to reliably detect (meaning >50%) loads <=20 cmH_2O during initial practice and performed below chance (<33%) on the IRDT. Participant 10: Excluded due to left mesial temporal SOZ with frequent interictal spikes (>10/min) in OFC, amygdala, and hippocampus. Participant 11: Excluded for inability to reliably detect loads <=25 cmH_2O during practice and performance below chance (<33%) on the IRDT.

	FEV1 (L)	FVC (L)	FEV1/FVC (%)	Raw ($cmH_2O/L/s$)	%Pred FEV1	%Pred FVC
P1	3.7	4.1	83.2	3.6	96.4	100.2
P2	3.6	4.5	84.8	4.2	92.2	97.7
P3	3.8	4.1	83.4	3.1	99.7	96.4
P4	4	4.1	82.8	4.4	91.6	95.6
P5	3.6	4.4	86.1	3.5	97.3	90.4
P6	3.6	3.6	84.2	3.8	88.6	93.8
P7	4	3.6	84.5	3.5	91.1	94.9
P8	3.8	4.1	82.8	5.7	97.2	101.6

Table S2. Pulmonary function tests.
Pulmonary function tests conducted prior to the IRDT task. FEV_1, forced expiratory volume in 1 s; FVC, forced vital capacity; Raw, airway resistance (measured via impulse oscillometry). Values reflect the highest of three FVC trials per participant. All participants had $FEV_1 > 70\%$ of predicted, indicating normal respiratory function (30).

REFERENCES

1. J. L. Feldman, The cardiorespiratory circuitry within the brainstem. *Eur. J. Neurosci. Suppl.* **3**, 171–180 (1990).

2. J. C. Smith, H. H. Ellenberger, K. Ballanyi, D. W. Richter, J. L. Feldman, Pre-Bötzinger complex: A brainstem region that may generate respiratory rhythm in mammals. *Science* **254**, 726–729 (1991).

3. J. L. Herrero, S. Khuvis, E. Yeagle, M. Cerf, A. D. Mehta, Breathing above the brain stem: Volitional control and attentional modulation in humans. *J. Neurophysiol.* **119**, 145–159 (2018).

4. M. Dutchman, T. E. Dick, The integrated brain network that controls respiration. *eLife* **12**, e83654 (2023).

5. J. L. Feldman, C. A. Del Negro, Breathing matters. *Nat. Rev. Neurosci.* **19**, 351–367 (2018).

6. C. F. Yang, J. L. Feldman, Efferent projections of excitatory and inhibitory preBötzinger complex neurons. *J. Comp. Neurol.* **526**, 1389–1402 (2018).

7. P. Trevizan-Baú, R. R. Dhingra, W. I. Furuya, D. Stanić, S. B. Mazzone, M. Dutschmann, Forebrain projection neurons target functionally diverse respiratory control areas in the midbrain, pons, and medulla oblongata. *J. Comp. Neurol.* **529**, 2243–2264 (2021).

8. H. H. Subramanian, G. Holstege, The midbrain periaqueductal gray changes the eupneic respiratory rhythm into a breathing pattern necessary for survival of the individual and of the species. *Prog. Brain Res.* **212**, 351–384 (2014).

9. K. Yackle, L. A. Schwarz, K. Kam, J. M. Sorokin, J. R. Huguenard, J. L. Feldman, M. A. Krasnow, Breathing control center neurons that promote arousal in mice. *Science* **355**, 1411–1415 (2017).

10. M. Deschênes, J. Moore, D. Kleinfeld, Sniffing and whisking in rodents. *Curr. Opin. Neurobiol.* **22**, 243–250 (2012).

11. W. Tan, S. Pagliardini, P. Yang, W. A. Janczewski, J. L. Feldman, Silencing preBötzinger complex somatostatin-expressing neurons induces persistent apnea in awake rat. *Nat. Neurosci.* **11**, 538–540 (2008).

12. B. J. Dlouhy, B. K. Gehlbach, C. J. Kreple, H. Kawasaki, H. Oya, C. Buzza, G. B. Richerson, Breathing inhibited when seizures spread to the amygdala and upon amygdala stimulation. *J. Neurosci.* **35**, 10281–10289 (2015).

13. X. Xu, X. Nie, W. Zhang, H.-H. Jiang, B. Liu, Y. Ren, T. Wang, X. Xu, J. Yang, F. Luo, A brainstem circuit controls cough-like airway defensive behaviors in mice. *eLife* **13**, RP102345 (2025).

14. J. Daubenmier, J. Sze, C. E. Kerr, M. E. Kemeny, W. Mehling, Follow your breath: Respiratory interoceptive accuracy in experienced meditators. *Psychophysiology* **50**, 777–789 (2013).

15. O. K. Harrison, L. Köchli, S. Marino, R. Luechinger, F. Hennel, K. Brand, K. E. Stephan, Interoception of breathing and its relationship with anxiety. *Neuron* **109**, 4080–4093 (2021).

16. K. M. Schottelkotte, S. A. Crone, Forebrain control of breathing: Anatomy and potential functions. *Front. Neurol.* **13**, 1041887 (2022).

17. M. Raux, C. Straus, S. Redolfi, C. Morelot-Panzini, A. Couturier, F. Hug, T. Similowski, Electroencephalographic evidence for pre-motor cortex activation during inspiratory loading in humans. *J. Physiol.* **578**, 569–578 (2007).

18. D. J. Eckert, P. G. Catcheside, J. H. Smith, P. A. Frith, R. D. McEvoy, Hypoxia suppresses symptom perception in asthma. *Am. J. Respir. Crit. Care Med.* **169**, 1224–1230 (2004).

19. M. S. Imtiaz, C. V. Bandoian, T. J. Santoro, Hypoxia driven opioid targeted automated device for overdose rescue. *Sci. Rep.* **11**, 24513 (2021).

20. Y. Kikuchi, S. Okabe, G. Tamura, W. Hida, M. Homma, K. Shirato, T. Takishima, Chemosensitivity and perception of dyspnea in patients with a history of near-fatal asthma. *N. Engl. J. Med.* **330**, 1329–1334 (1994).

21. P. W. Davenport, Y. Kifle, Inspiratory resistive load detection in children with life-threatening asthma. *Pediatr. Pulmonol.* **32**, 44–48 (2001).

22. W.-j. Guan, Z.-y. Ni, Y. Hu, W.-h. Liang, C.-q. Ou, J.-x. He, L. Liu, H. Hong, Y. Liu, H. Chen, G. Guo, R. Zheng, S. Qiu, J. Luo, C. Ye, S. Zhu, L. Cheng, T. Ye, S. Li, Z. Zheng, S. Zhong, H. Li, L. Tao, Y. Li, Z. Xiang, Y. Wu, C. Liu, R. Li, P. Jin, C. Lang, X. Ma, H. Deng, J. Ren, C. Zhong, Y. Zhan, S. Qian, R. Li, C. Jin, L. Lu, G. Wang, Z. Liu, Y. Chen, C. Liang, Q. Tang, P. Wang, Z. Li, J. Han, S. Qiu, C. Li, L. Li, K. Ou, B. Chen, B. Chen, Clinical characteristics of coronavirus disease 2019 in China. *N. Engl. J. Med.* **382**, 1708–1720 (2020).

23. T. Ritz, Probing the psychophysiology of the airways: Physical activity, experienced emotion, and facially expressed emotion. *Psychophysiology* **41**, 809–821 (2004).

24. A. von Leupoldt, P. Y. S. Chan, M. M. Bradley, P. J. Lang, P. W. Davenport, The impact of anxiety on the neural processing of respiratory sensations. *Neuroimage* **55**, 247–252 (2011).

25. L. Genecand, M. Altarelli, A. Binkova, S. Loew, S. Vaudan, G. Gex, I. Frésard, Dysfunctional breathing symptoms, functional impact and quality of life in patients with long COVID-19: A prospective case series. *BMJ Open Respir. Res.* **10**, e001803 (2023).

26. A. von Leupoldt, B. Dahme, Cortical substrates for the perception of dyspnea. *Chest* **128**, 345–354 (2005).

27. N. K. Burki, L.-Y. Lee, Mechanisms of dyspnea. *Chest* **138**, 1196–1201 (2010).

28. N. Nikolova, O. Harrison, S. Toohey, M. Brændholt, N. Legrand, C. Correa, M. Vejlø, M. S. Jensen, F. Fardo, M. Allen, The respiratory resistance sensitivity task: An automated method for quantifying respiratory interoception and metacognition. *Biol. Psychol.* **170**, 108325 (2022).

29. M. I. M. Noble, H. L. Frankel, W. Else, A. Guz, The ability of man to detect added resistive loads to breathing. *Clin. Sci.* **41**, 285–287 (1971).

30. N. R. Bhakta, A. McGowan, K. A. Ramsey, B. Borg, J. Kivastik, S. L. Knight, K. Sylvester, F. Burgos, E. R. Swenson, K. McCarthy, B. G. Cooper, F. García-Río, G. Skloot, M. McCormack, C. Mottram, C. G. Irvin, I. Steenbruggen, A. L. Coates, D. A. Kaminsky, European Respiratory Society/American Thoracic Society technical statement: Standardisation of the measurement of lung volumes, 2023 update. *Eur. Respir. J.* **62**, 2301519 (2023).

31. X. Navarro-Sune, M. Raux, A. L. Hudson, T. Similowski, M. Chavez, Cycle-frequency EEG analysis improves assessment of respiratory-related cortical activity. *Physiol. Meas.* **45**, 095003 (2024).

32. A. L. Hudson, M. C. Niérat, M. Raux, T. Similowski, The relationship between respiratory-related premotor potentials and small perturbations in ventilation. *Front. Physiol.* **9**, 621 (2018).

33. M. Georges, E. Morawiec, M. Raux, J. Gonzalez-Bermejo, P. F. Pradat, T. Similowski, C. Morélot-Panzini, Cortical drive to breathe in amyotrophic lateral sclerosis: A dyspnoea-worsening defense? *Eur. Respir. J.* **47**, 1818–1828 (2016).

34. O. K. Faull, H. H. Subramanian, M. Ezra, K. T. Pattinson, The midbrain periaqueductal gray as an integrative and interoceptive neural structure for breathing. *Neurosci. Biobehav. Rev.* **98**, 135–144 (2019).

35. B. R. Kaada, Somato-motor, autonomic and electrocorticographic responses to electrical stimulation of rhinencephalic and other structures in primates, cat, and dog; a study of responses from the limbic, subcallosal, orbito-insular, piriform and temporal cortex, hippocampus-fornix and amygdala. *Acta Physiol. Scand. Suppl.* **24**, 1–262 (1951).

36. K. H. Pribram, M. A. Lennox, R. H. Dunsmore, Some connections of the orbito-fronto-temporal, limbic and hippocampal areas of *Macaca mulatta*. *J. Neurophysiol.* **13**, 127–135 (1950).

37. S. W. Centanni, A. C. Janes, D. L. Haggerty, B. Atwood, F. W. Hopf, Better living through understanding the insula: Why subregions can make all the difference. *Neuropharmacology* **198**, 108765 (2021).

38. Z. Chen, F. L. Eldridge, P. G. Wagner, Respiratory-associated thalamic activity is related to level of respiratory drive. *Respir. Physiol.* **90**, 99–113 (1992).

39. A. Sheriff, G. Zhou, V. Sagar, C. Zelano, Breathing orchestrates synchronization of sleep oscillations in the human hippocampus. *Proc. Natl. Acad. Sci. U.S.A.* **121**, e2405395121 (2024).

40. A. K. Seth, Interoceptive inference, emotion, and the embodied self. *Trends Cogn. Sci.* **17**, 565–573 (2013).

41. A. K. Seth, K. Suzuki, H. D. Critchley, An interoceptive predictive coding model of conscious presence. *Front. Psychol.* **2**, 395 (2012).

42. M. Allen, F. Fardo, M. J. Dietz, H. Hillebrandt, K. J. Friston, G. Rees, A. Roepstorff, Anterior insula coordinates hierarchical processing of tactile mismatch responses. *Neuroimage* **127**, 34–43 (2016).

43. X. Wang, Q. Wu, L. Egan, X. Gu, P. Liu, H. Gu, J. Fan, Anterior insular cortex plays a critical role in interoceptive attention. *eLife* **8**, e42265 (2019).

44. C. Padoa-Schioppa, J. A. Assad, Neurons in the orbitofrontal cortex encode economic value. *Nature* **441**, 223–226 (2006).

45. E. B. Knudsen, J. D. Wallis, Taking stock of value in the orbitofrontal cortex. *Nat. Rev. Neurosci.* **23**, 428–438 (2022).

46. N. Floyd, J. L. Price, A. T. Ferry, K. A. Keay, R. Bandler, Orbitomedial prefrontal cortical projections to distinct longitudinal columns of the periaqueductal gray in the rat. *J. Comp. Neurol.* **422**, 556–578 (2000).

47. M. Raux, L. Tyvaert, M. Ferreira, F. Kindler, E. Bardinet, C. Karachi, C. Morelot-Panzini, C. Lambermont, D. L. Cohen, J.-C. Alvarez, T. Similowski, Functional magnetic resonance imaging suggests automatization of the cortical response to inspiratory threshold loading in humans. *Respir. Physiol. Neurobiol.* **189**, 571–580 (2013).

48. N. Nikolova, J. F. Ehmsen, L. Banellis, M. Brændholt, C. Steenkjær, M. Vejlø, M. G. Allen, Microstructural brain correlates of inter-individual differences in respiratory interoception. *J. Neurosci.* **45**, e0787242025 (2025).

49. A. L. Hudson, M.-E. Day, M. T. Williams, O. K. Harrison, The potential role of the anterior insular cortex and interoception on dyspnea in chronic obstructive pulmonary disease. *Respir. Physiol. Neurobiol.* **336**, 104441 (2025).

50. C. Zelano, H. Jiang, G. Zhou, N. Arora, S. Schuele, J. Rosenow, J. A. Gottfried, Nasal respiration entrains human limbic oscillations and modulates cognitive function. *J. Neurosci.* **36**, 12448–12467 (2016).

51. M. Allen, A. Levy, T. Parr, K. J. Friston, In the body's eye: The computational anatomy of interoceptive inference. *PLoS Comput. Biol.* **18**, e1010604 (2022).

52. J. H. Silverman, R. P. Andersen, The efficiency of the mouthpiece and noseclip in preventing air leakage during pulmonary function tests. *Am. Rev. Tuberc.* **77**, 538–540 (1958).

53. D. M. Groppe, S. Bickel, A. R. Dykstra, X. Wang, P. Mégevand, M. R. Mercier, F. A. Lado, A. D. Mehta, C. J. Honey, A. D. Norden, iELVis: An open source MATLAB toolbox for localizing and visualizing human intracranial electrode data. *J. Neurosci. Methods* **281**, 40–48 (2017).

54. S. M. Smith, Fast robust automated brain extraction. *Hum. Brain Mapp.* **17**, 143–155 (2002).

55. M. Jenkinson, S. Smith, A global optimisation method for robust affine registration of brain images. *Med. Image Anal.* **5**, 143–156 (2001).

56. X. Papademetris, M. P. Jackowski, N. Rajeevan, M. DiStasio, H. Okuda, R. T. Constable, L. H. Staib, BioImage suite: An integrated medical image analysis suite: An update. *Insight J.* **2006**, 209 (2006).

57. B. Fischl, FreeSurfer. *Neuroimage* **62**, 774–781 (2012).

58. M. R. Mercier, S. Bickel, P. Megevand, D. M. Groppe, C. E. Schroeder, A. D. Mehta, F. A. Lado, Evaluation of cortical local field potential diffusion in stereotactic electro-encephalography recordings: A glimpse on white matter signal. *Neuroimage* **147**, 219–232 (2017).

59. R. Janca, P. Jezdik, R. Cmejla, M. Tomasek, G. A. Worrell, M. Stead, J. Wagenaar, Detection of interictal epileptiform discharges using signal envelope distribution modelling: Application to epileptic and non-epileptic intracranial recordings. *Brain Topogr.* **28**, 172–183 (2015).

60. S. Ray, J. H. R. Maunsell, Different origins of gamma rhythm and high-gamma activity in macaque visual cortex. *PLoS Biol.* **9**, e1000610 (2011).

61. G. Buzsáki, C. A. Anastassiou, C. Koch, The origin of extracellular fields and currents—EEG, ECoG, LFP and spikes. *Nat. Rev. Neurosci.* **13**, 407–420 (2012).

62. Y. Benjamini, Y. Hochberg, Controlling the false discovery rate: A practical and powerful approach to multiple testing. *J. R. Stat. Soc. Series B Stat. Methodol.* **57**, 289–300 (1995).

63. J. Noto, J. Nguyen, A. N. Schachinger, M. J. Rieger, BreathMetrics: A MATLAB toolbox for analyzing breathing waveforms. *Front. Physiol.* **9**, 785 (2018).

64. M. Dhamala, H. Liang, S. L. Bressler, M. Ding, Granger-Geweke causality: Estimation and interpretation. *Neuroimage* **175**, 460–463 (2018).

65. A. K. Seth, A. B. Barrett, L. Barnett, Granger causality analysis in neuroscience and neuroimaging. *J. Neurosci.* **35**, 3293–3297 (2015).

66. M. Dhamala, G. Rangarajan, M. Ding, Analyzing information flow in brain networks with nonparametric Granger causality. *Neuroimage* **41**, 354–362 (2008).

67. A. B. L. Tort, R. Komorowski, H. Eichenbaum, N. Kopell, Measuring phase-amplitude coupling between neuronal oscillations of different frequencies. *J. Neurophysiol.* **104**, 1195–1210 (2010).

68. P. Rajasethupathy, S. Sankaran, J. H. Marshel, C. K. Kim, E. Ferenczi, S. Y. Lee, A. Berndt, Y. Ramakrishnan, J. J. J. Kane, K. Deisseroth, Projections from neocortex mediate top-down control of memory retrieval. *Nature* **526**, 653–659 (2015).

69. M. Siegel, T. J. Buschman, E. K. Miller, Cortical information flow during flexible sensorimotor decisions. *Science* **348**, 1352–1355 (2015).

70. A. M. Bastos, J. Vezoli, C. A. Bosman, J.-M. Schoffelen, R. Oostenveld, J. R. Dowdall, P. De Weerd, H. Kennedy, P. Fries, Visual areas exert feedforward and feedback influences through distinct frequency channels. *Neuron* **85**, 390–401 (2015).

71. A. Thiele, A. Pooresmaeili, L. S. Delicato, J. L. Herrero, P. R. Roelfsema, Additive effects of attention and stimulus contrast in primary visual cortex. *Cereb. Cortex* **19**, 2970–2981 (2009).
